# Time-Resolved Resonant Inelastic X‑ray Scattering Reveals How Orbital Symmetry Alignment Enables C–H Activation

**DOI:** 10.1021/jacs.6c05747

**Published:** 2026-04-30

**Authors:** Timo Dederichs, Ambar Banerjee, Victoria Kabanova, Robert Stefanuik, Antonia Freibert, Emma V. Beale, Florian Dworkowski, Rebeca G. Castillo, Philip J. M. Johnson, Claudio Cirelli, Nils Huse, Camila Bacellar, Raphael M. Jay, Philippe Wernet

**Affiliations:** † Department of Physics and Astronomy, 8097 Uppsala University, 75120 Uppsala, Sweden; ‡ Research Institute for Sustainable Energy (RISE), 637829 TCG Centres for Research and Education in Science and Technology (TCG-CREST), Kolkata 700091, India; § Department of Physics, 14915 University of Hamburg, 22761 906 Hamburg, Germany; ∥ Paul-Scherrer Institute, CH-5232 Villigen, Switzerland; ⊥ Laboratory of Ultrafast Spectroscopy, Ecole Polytechnique Federale de Lausanne (EPFL), CH-1015 Lausanne, Switzerland

## Abstract

Photochemical C–H activation reactions mediated by transition metal complexes often proceed via the formation of σ-complexes. In these intermediates, the metal center coordinates to a C–H σ-bond, and metal–ligand donation and back-donation interactions ultimately lead to C–H bond cleavage. Because metal–alkane σ-complexes are weakly bond and short-lived, current experimental methods provide limited access to the transient electronic-structure effects that control their reactivity. Here, we photochemically prepared three different types of Rh–alkane σ-complexes in solution and demonstrate how optical pump and X-ray probe spectroscopy gives access to their decisive valence-electron interactions. With femtosecond-resolution and Rh specific X-ray absorption spectroscopy (XAS) and resonant inelastic X-ray scattering (RIXS) at the Rh L_3_-edge we access the orbital interactions between the Rh centers, the ancillary ligands and the alkane C–H σ-bonds of the Rh–alkane σ-complexes. Supported by theoretical calculations, we identify spectral fingerprints of Rh-alkane donation and back-donation interactions and find trends for the reactivity of the σ-complexes toward C–H activation. We uncover the particular importance of specific occupied molecular orbitals with specific symmetry in modulating reactivity by providing, channeling and directing electron density via the metal to and from the C–H bond. Elucidating the electronic factors that facilitate C–H bond activation provides a basis for future ligand substitutions aimed at enhancing the reactivity of metal-alkane σ-complexes with enhanced efficiency of the activation step.

## Graphical abstract

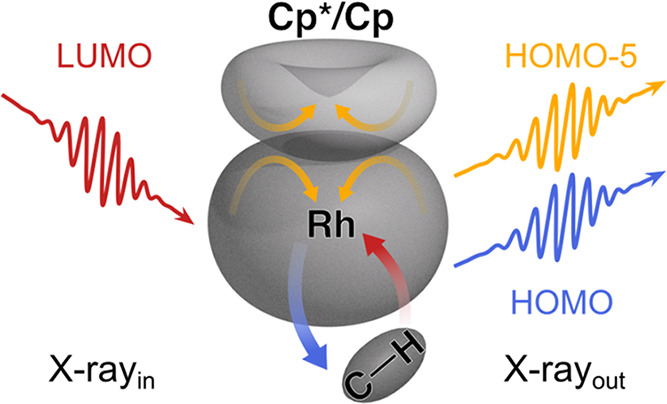

## Introduction

Being able to selectively activate and transform a C–H bond, one of the most ubiquitous albeit inert bonds in nature, has been a long-standing goal of organic and metal–organic chemistry. C–H functionalization in alkanes, in particular, opens the door to streamlined synthesis without the need for prefunctionalized starting materials and multistep syntheses.^1–4^ Shorter and more efficient synthetic routes reduce waste and improve sustainability, which are important goals of green chemistry.^5–7^ While significant progress has been made, numerous challenges in realizing the very first step of C–H functionalization reactions, the mere activation of C–H bonds under mild conditions, still hinder applications.

One approach to activating C–H bonds in alkanes without excessive use of energy is using photoexcited transition metal complexes.^8,9^ Such reactions can be performed in room-temperature alkane solutions and start with the dissociation of one or more ligands from the excited transition metal complex, generating a low-valent reactive metal complex.^10,11^ The open site at the metal center allows coordination of an alkane^12–15^ and, eventually, activation of one of its C–H bonds.^16–19^ The essential intermediates in these photochemical C–H activation reactions are so-called σ-alkane complexes (short σ-complexes), where a C–H bond loosely binds to the metal center. σ-complexes are short-lived with kinetically stable conformers thought to occur on time scales in the range of picoseconds.^20,21^ The electronic properties of a σ-complex critically determines if the initial metal complex is suitable for C–H activation: The reaction may proceed toward C–H activation by oxidative addition, where the metal is inserted into the C–H bond and formally oxidized, or it may stop when the σ-complex has formed. Hence, reactivity in photochemical C–H activation by oxidative addition critically depends on the metal-alkane bonding in the σ-complex. Of the established trends for correlating properties and reactivity of σ-complexes, the “electron richness”^8^ of the metal center and thereby its propensity for being oxidized, have been identified as being decisive for activation.^8^ As in other mechanisms of C–H activation by transition metal complexes,^22–25^ the “electron richness” of the metal center is determined by the orbital interactions with its ligands. The interactions that are thought to ultimately help breaking the C–H bond are those between the metal d orbital and the σ/σ*-orbitals of the coordinating C–H bond. According to the conceptual picture established by Saillard and Hoffmann,^26^ these metal-C–H interactions can be described by donation from the C–H σ-orbital to a metal d-orbital (alkane-to-metal donation) and, in parallel, by back-donation from a metal d-orbital to the C–H σ*-orbital (metal-to-alkane donation).^27,28^ This synergistic bonding scheme is essential for enabling C–H activation, as it determines both the coordination of the C–H bond to the metal center and the weakening of the C–H bond. Thus, probing these interactions in short-lived σ-complexes is crucial for understanding what determines their reactivity toward C–H activation.

Isotope labeling^12^ and NMR spectroscopy^29–31^ have been instrumental for revealing overall mechanisms and reactivities in C–H activation. Neutron and X-ray diffraction^32–35^ were decisive for determining structures of freeze-trapped or crystallized σ-complexes. Time-resolved infrared (IR) spectroscopy has proven essential in detecting and structurally characterizing short-lived reaction intermediates in the course of the reaction.^10,18,19,36–40^ All these established methods, however, lack direct sensitivity to the local valence electronic structure and are therefore limited in their ability to correlate the hypothesized metal-C–H interactions with reactivity toward C–H activation.^26^

We have previously used time-resolved X-ray absorption spectroscopy (XAS) at the Rh and Cr L-edges to study CpRh­(CO)-alkane^20,41^ and Cr­(CO)_5_-alkane^21^ σ-complexes (alkane = octane). However, XAS probes only the unoccupied valence electronic structure and is therefore sensitive only to the unoccupied antibonding part of the molecular orbital interactions in σ-complexes. To obtain more comprehensive orbital information, to test the concepts of Hoffmann,^26^ and to potentially relate orbital interactions to reactivity trends in C–H activation,^8^ the occupied valence electronic structure must be probed as well. This capability is provided by so-called valence-to-core (VtC) resonant inelastic X-ray scattering (RIXS),^42–44^ which is the X-ray analog of resonance Raman scattering. Metal L-edge VtC RIXS probes valence excitations in the form of an energy transfer to the molecules by the scattered X-rays.^39–44^ It thereby gives access to valence transitions that are hard or impossible to access in optical absorption spectroscopy because they are dipole-forbidden or masked by the solvent response.^39,40,46–48^

In this study, we use a combination of time-resolved XAS and VtC RIXS at the Rh L_3_-edge in the so-called tender X-ray range at around 3000 eV. The RIXS part represents, to the best of our knowledge, the first results from time-resolved RIXS experiments on a 4d metal complex. For the systems studied here (Figure 1a), Rh L-edge RIXS can be approximated by a two-step process with X-ray absorption (XAS in Figure 1b) followed by X-ray emission (XES in Figure 1b), and it can be approximately interpreted within the one-electron orbital energy picture (Figure 1b). Within this picture, a Rh 2p core electron is promoted to unoccupied molecular orbitals in the XAS step and in the subsequent XES step, the Rh 2p core hole is filled by a valence electron from an occupied orbital. VtC RIXS at the Rh L_3_-edge can thus be described as probing one-electron transitions between the occupied and unoccupied valence molecular orbitals. The corresponding valence-excited final states makes the method locally sensitive to the valence electronic structure at the Rh center.^40^ In addition, both the XAS and the XES steps are subject to dipole selection rules (Δ*l* = ±1) thereby predominantly probing p → d transitions in XAS and d → p transitions in XES. This sequence defines the sensitivity of the probe to d-d valence excitations where a given energy transfer ΔE corresponds to the transition energy of a given d-d excitation.^39,40^ Chemical bonding and ligand coordination become accessible by sensitivity to changes in orbital overlap and orbital mixing locally at the Rh atom.^41^

**Figure 1:**
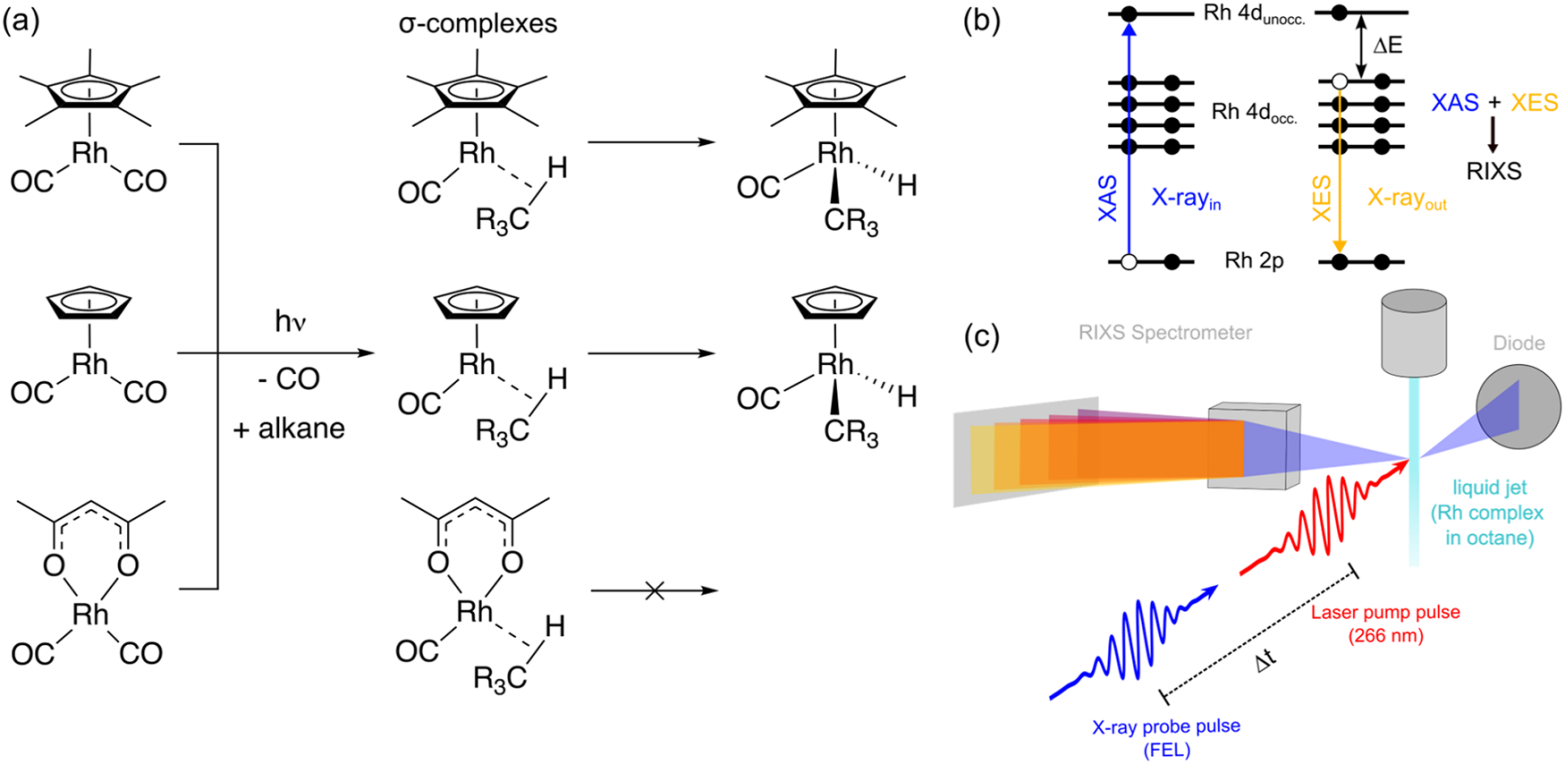
(a) Simplified schemes of the photoinduced reactions studied here of Cp*Rh­(CO)_2_, CpRh­(CO)_2_, and Rh­(acac)­(CO)_2_ (top to bottom) in alkane solution with CO to alkane ligand substitution for all complexes and C–H activation for Cp*Rh­(CO)_2_ and CpRh­(CO)_2_. (b) Schematic representation of the Valence-to-Core (VtC) resonant inelastic X-ray scattering (RIXS) process at the Rh L-edge (XAS = X-ray absorption spectroscopy, XES = X-ray emission spectroscopy, Δ*E* = energy transfer). (c) Schematic of the experiment with a UV laser pump pulse triggering the photochemical reaction in a liquid jet and a subsequent X-ray probe pulse from a free electron laser (FEL, the Swiss free-electron laser SwissFEL in our case), probing the valence electronic structure as a function of time delay Δ*t* between UV and X-ray pulses with XAS using a diode and RIXS using a spectrometer.

We used time-resolved Rh L_3_-edge VtC RIXS at the Swiss X-ray free-electron laser (SwissFEL, Figure 1c) to characterize the valence electronic structure in the short-lived Rh-alkane σ-complexes Cp*Rh­(CO)-octane, CpRh­(CO)-octane, and Rh­(acac)­(CO)-octane (where Cp = cyclopentadienyl, Cp* = methylated Cp, acac = acetylacetonate, see Figure 1a). We find that changes in the molecular-orbital interactions between the Rh center, the C–H bond and the ancillary ligands in the studied σ-complexes correlate with the propensity of the metal complex to activate the C–H bond. By extending established notions of “electron richness” and oxidation of the metal center as well as donation and back-donation interactions, we arrive at a new description of how the alignment of molecular orbital symmetries provides the necessary electron density for efficient orbital interactions in metal-mediated C–H activation by oxidative addition.

## Methods

### Materials

The Cp*Rh­(CO)_2_ and CpRh­(CO)_2_ samples were purchased from HetCat, Rh­(acac)­(CO)_2_ and the solvent octane (reagent grade) from Sigma-Aldrich. All Cp*Rh­(CO)_2_ and Rh­(acac)­(CO)_2_ samples were prepared as 20 mM solutions in octane. CpRh­(CO)_2_ was prepared as 30 mM solution in octane.

### Experimental Details

The experimental setup at the Alvra experimental station of SwissFEL is schematically depicted in Figure 1c. Samples were delivered to the experimental chamber by a cylindrical liquid jet with a diameter of 75 μm, collected in a catcher system underneath the interaction point and recirculated to the sample reservoir. The sample integrity was checked by recording UV/vis spectra before and after exposure to the X-rays, as well as by inspecting the steady-state X-ray absorption spectrum. No spectral changes were detected during the experiment, but sample batches were nevertheless replaced every ∼8h. To minimize X-ray scattering noise and enhance X-ray transmission, the experimental chamber was kept under 500 mbar He atmosphere. The samples were excited using 266 nm light (third harmonic of an 800 nm Ti:sapphire amplified laser system with a pulse duration of ∼75 fs) at a repetition rate of 50 Hz and a laser fluence of 16.5 mJ/cm^2^ (36.8 mJ/cm^2^ for RIXS of Cp*Rh­(CO)_2_). We verified that the observed pump–probe effects varied linearly with UV laser fluence (see Figure S1 in SI). A detailed discussion about potential thermal effects is given in the SI in section S1.3. Based on previous estimations, the quantum yield for CO dissociation is ∼4% for CpRh­(CO)_2_ and ∼7% for Rh­(acac)­(CO)_2_.^20^ In the case of Cp*Rh­(CO)_2_, it was reported to be a few percent and our experiments confirmed that order of magnitude.^49^

For X-ray absorption spectra, the incidence photon energy was tuned across the Rh L_3_-edge between 2995 and 3015 eV using a Si(111) monochromator with a bandwidth of ∼0.4 eV. The temporal duration of the X-rays was determined to be ∼40 fs. At the interaction point, the X-ray spot size was 20 × 20 μm^2^ (FWHM), and the laser spot size was ∼60 × 80 μm^2^ (FWHM). X-ray absorption data were recorded using an avalanche photo diode in total florescence yield (TFY) mode. The fluorescence signal was normalized to the *I*_0_ intensity of the monochromatized X-ray beam measured upstream the beam path. The FEL was running at a repetition rate of 100 Hz (twice the optical laser repetition rate) allowing for measurements of consecutive laser-on and laser-off spectra which were used for generating the difference signals. Absorption spectra and delay traces were acquired by collecting the X-ray fluorescence over 500 or 1000 pulses per measurement point (2500 for delay traces measured up to 100 ns). The measured signals were normalized to I_0_ on a pulse-to-pulse basis. The temporal resolution of our experiment amounted to 270 fs (Gaussian FWHM, the experimental factors determining the temporal resolution are detailed in Section S2.1.3 in the Supporting Information).

The Rh VtC L_3_-RIXS data were recorded using a von-Hamos type X-ray emission spectrometer with one 250 mm cylindrical bent Si(111) segmented crystal and a JUNGFRAU 4.5 M detector. The energy transfer axis was calibrated for each sample individually by fitting the position of the elastic line and setting it to 0 eV. The combined energy resolution was determined to ∼0.75 eV (FWHM) by fitting the elastic line below the absorption onset at 2995 eV using a Gaussian (with the monochromator bandwidth of ∼0.4 eV, FWHM, the spectrometer bandwidth amounted to ∼0.6 eV for X-ray fluorescence emission). Each pixel of the raw single-shot JUNGFRAU detector images was corrected for both pedestal offsets and gain factors prior to extracted energy in keV. To reduce noise and exclude unphysical counts, energy thresholds of 2.0 keV (low) and 10.0 keV (high) were applied on a per-pixel basis. For additional S/N improvement, two regions of interest (ROIs) were defined on the 2D detector and subtracted from each other. The first ROI (signal) was positioned around the VtC features. The second ROI (background) was placed at the same position along the dispersive axes but offset along the nondispersive axis. The VtC RIXS data were acquired by collecting 1000 or 2000 pulses per measurement point.

### Computational Details

The quantum chemical computations were performed using the ORCA 5.0.2 program package.^50^ All geometries were optimized at the TPSSh/def2-TZVP level of theory^51,52^ and checked for absent imaginary normal modes by frequency computations, confirming that the optimized structures correspond to energetic minima on the reaction pathway. To account for solvent effects in all calculations, the CPCM polar continuum model^53^ was used, with hexane as the solvent. The Rh L_3_-edge XAS and RIXS were simulated following closely the protocol adopted for predicting similar CpRh­(CO)_2_, Rh­(acac)­(CO)_2_ and associated intermediates by Banerjee, Jay et al.^41^ The calculations employed the restricted orbital subspace TD-DFT method^54^ within the Tamm-Damcoff approximation.^55^ To account for second order relativistic effects, the ZORA method was used.^56^ The SARC-ZORA-TZVPP basis set was used for the Rh atom and the ZORA-def2-TZVPP basis set for the rest of the atoms.^57^ To speed up the computations, the RIJCOSX^58^ protocol was adopted with the auxiliary def2-TZVP/C basis set.

To calculate the activation barrier for C–H activation, the protocol established by Jay, Banerjee et al.,^20^ for CpRh­(CO)_2_ and Rh­(acac)­(CO)_2_ was adopted for Cp*Rh­(CO)_2_. The minimum energy reaction path from the σ-complex to the corresponding C–H activated product was located using the Nudged-Elastic-Band (NEB) method.^59^ Subsequently, the geometry at the climbing image (CI) along the NEB pathway was used as an initial guess for the optimization of the transition state structure between the σ-complex and the corresponding C–H activated product. The transition state was found to have one imaginary normal mode. The single point energies of all, the dicarbonyl species, the respective σ-complex, the transition state structure, and the C–H activated species, were computed at the DLPNO–CCSD­(T)­(TightPNO)/def2-TZVPP/CPCM­(hexane)^60^ level of theory. The free energy corrections for all species were obtained from a frequency calculation at the TPSSh/def2-TZVP/CPCM­(hexane) level of theory. To compare the free-energy profiles of the three systems, Cp*Rh­(CO)_2_/CpRh­(CO)_2_/Rh­(acac)­(CO)_2_ + octane were set as the reference points point from which the free energy differences are computed from. The choice of the actual reactants as the reference points is justified as they represent the lowest point in the free energy landscape and are stable until irradiated with a UV laser pulse.

For the XAS spectra, 100 states were computed as excited from the three 2p orbitals of the Rh center, covering the entire L_3_-edge spectrum. Excitation energies and oscillator strengths obtained from the calculations were convoluted using a Voigt profile combining a 1.9 eV Lorentzian (FWHM), which accounts for the Rh 2p core-hole lifetime broadening,^61^ and a 0.6 eV Gaussian (FWHM) to account for the combined effect of the incident photon energy bandwidth (0.4 eV) and conformational broadening. To correct for the effects of core-hole relaxation, a uniform energy shift of −22.25 eV was applied to all computed spectra. The shift was determined by maximizing the calculated cross-correlation between the experimental and calculated spectra for each complex. Applying this shift resulted in an accurate match between theory and experiment. Difference spectra were generated by subtracting the convoluted spectrum of the respective parent complex from the convoluted spectrum of a given intermediate species.

For RIXS spectra, the three 2p orbitals of Rh were rotated into a restricted subspace, consisting of 20 occupied and 20 unoccupied orbitals. Core-excited (intermediate) and valence-excited (final) states were computed by promoting electrons from the occupied orbital subspace (20 + 3 orbitals) into the 20 selected unoccupied orbitals. To cover transitions in the energy transfer range of 0–16 eV, 400 valence-excited states were solved as well as 60 core-excited states for all species. The transition dipole moments for excitations from the ground state to the core-excited state and decay from core-excited states to valence-excited states were calculated using the Multi-Wfn 3.6 program package.^62^ RIXS intensities were calculated using the Kramers-Heisenberg formula and subsequently convoluted along the incident energy axis, analogous to the XAS spectra, using a Voigt profile combining a 1.9 eV Lorentzian (Rh 2p core-hole lifetime broadening), and a 0.6 eV Gaussian (FWHM, for incidence energy bandwidth and conformational broadening along the incidence photon energy axis). Along the energy transfer axis, the intensities were broadened with a 1.3 eV Gaussian (FWHM, combined spectrometer bandwidth of 0.6 eV and conformational broadening, for the emission axis of the RIXS spectra a larger Gaussian width for conformational broadening had to be chosen compared to the XAS spectra because the RIXS final states turned out to be more sensitive to conformational changes). Details about how the calculated RIXS spectra were extracted from calculated RIXS maps or planes are described in the SI (see Section S2.3.7). Consistent with the computed XAS spectra, a uniform shift of the −22.25 eV of the incidence photon energy was applied to all RIXS spectra. Difference RIXS spectra were generated by subtracting the convoluted spectrum of the respective parent complex from the convoluted spectrum of a given intermediate species.

## Results

In Figure 2 we present the steady-state and transient Rh L_3_-edge X-ray absorption spectra (top) as well as the steady-state and time-resolved Rh L_3_-edge RIXS spectra (bottom) of Cp*Rh­(CO)_2_, CpRh­(CO)_2_, and Rh­(acac)­(CO)_2_ in octane solution. In analogy to our previous assignments,^20^ all dicarbonyl species Cp*Rh­(CO)_2_, CpRh­(CO)_2_, and Rh­(acac)­(CO)_2_ exhibit two dominant XAS peaks (Figure 2, top) that are attributable to the same transitions: The first peak or shoulder at ∼3006 eV arises from excitation of a Rh 2p core electron to the lowest unoccupied molecular orbital (LUMO), which is derived from the empty 4d-orbital of the Rh­(I) d^8^ ground state configuration (see excitation Scheme Figure 1(b)). The second peak at ∼3007.5 eV is attributed to the transitions of Rh 2p core electrons into higher-lying unoccupied orbitals featuring mainly CO and/or Cp*/Cp/acac ligand character. Metal-to-ligand back-donation imparts Rh 4d character to these ligand-dominated orbitals and facilitates access to them by p → d dipole transitions.

**Figure 2:**
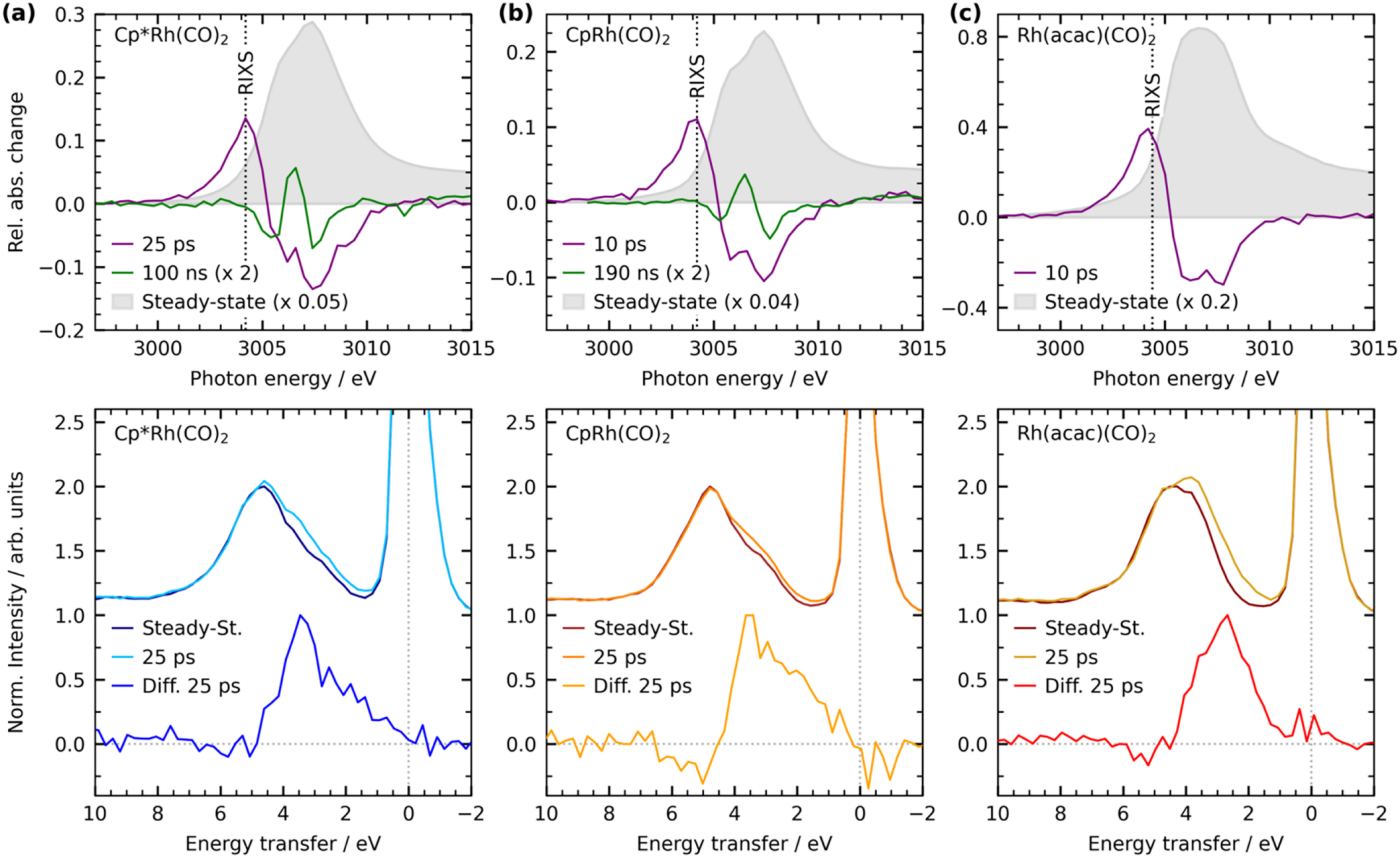
(Top) Steady-state and transient Rh L_3_-edge X-ray absorption spectra of (a) Cp*Rh­(CO)_2_, (b) CpRh­(CO)_2_ and (c) Rh­(acac)­(CO)_2_ in octane solution at different pump–probe delays (edge-jumps of the steady-state spectra, i.e., intensities at 3015 eV, are normalized to 1, steady-state spectra are scaled individually for illustration, difference spectra are plotted relative to the edge-jumps, dotted lines at 3004.2 eV for Cp*Rh­(CO)_2_ and CpRh­(CO)_2_ and at 3004.4 eV for Rh­(acac)­(CO)_2_ indicate the photon energy at which Rh L_3_-edge RIXS spectra were measured, see Figure 2 (bottom)). The spectra of CpRh­(CO)_2_ and the 10 ps spectrum of Rh­(acac)­(CO)_2_ are reproduced from our previous publication.^20^ (Bottom) Steady-state and transient VtC Rh L_3_-edge RIXS spectra of (a) Cp*Rh­(CO)_2_, (b) CpRh­(CO)_2_ and (c) Rh­(acac)­(CO)_2_ in octane solution as measured at pump probe delays of 25 ps. The steady-state RIXS spectra were normalized to their maximum at the most intense inelastic feature (intensities at 4.8 eV) and the same scaling factor was applied to the transient RIXS spectra for direct comparison of relative intensities. The difference spectra (25 ps spectra minus steady-states spectra) are normalized to their respective maximum at the most intense inelastic feature (intensities at 3.5 eV in Cp*Rh­(CO)_2_ and CpRh­(CO)_2_ and 2.5 eV in Rh­(acac)­(CO)_2_).^20^

Upon electronic excitation by the UV pump pulse, a pre-edge peak emerges in all three complexes at ∼3004 eV (see the transient difference XAS spectra at 10 and 25 ps). The time evolution of this feature is detailed by the delay traces in the SI (Figure S4).^20,21^ Guided by our previous study of CpRh­(CO)_2_ ^20^ and based on kinetic fits (see SI section S2.1) we assign the transient difference XAS spectra at 10 and 25 ps (Figure 2, top) to the three alkane σ-complexes Cp*Rh­(CO)-octane, CpRh­(CO)-octane, and Rh­(acac)­(CO)-octane (an assignment justified a posteriori by our calculations, see Figure 3). The lower pre-edge energies in the σ-complexes (3004.2 eV) compared to the dicarbonyl complexes (3006 eV) is a result of substituting the strongly interacting CO ligand with the weakly interacting C–H bond of an octane molecule. This ligand exchange destabilizes the LUMOs of the σ-complexes and hence decreases the 2p → LUMO transition energies. The same trend was previously observed in similar ligand substitution reactions of other transition metal complexes.^21,40,63,64^

To investigate the occupied valence electrons, we present the steady-state and time-resolved Rh L_3_-edge RIXS spectra of the three Rh dicarbonyl and σ-complexes in Figure 2 (bottom), recorded at the pre-edge peaks of the σ-complex XAS spectra (dotted lines in the Figure 2, top). For experimental simplicity, the same incident energies were chosen for both the σ-complexes and the intact ground-state dicarbonyl species, even though these energies are off resonance in the dicarbonyl species. This choice is justified for the present analysis, since the off-resonance XAS transitions in the dicarbonyl species still probe their Rh 2p → LUMO transitions. All three time-resolved RIXS spectra were recorded at a pump–probe delay of 25 ps where populations of the σ-complexes are maximal in all three cases (see delay traces in SI, Figure S4).

All observed RIXS features can predominantly be assigned to d-d valence-excited final states. Within our one-electron orbital energy approximation, the XAS step generates Rh 2p core holes via Rh 2p → 4d-derived LUMO transitions in the pre-edge. These 2p core holes are then filled in the subsequent XES step by electrons form occupied Rh 4d-derived orbitals, leading to d-d excited final states (Figure 1b). For the dicarbonyl complexes, the RIXS spectra consist of a broad band of inelastic scattering intensities in the energy transfer region from 2 to 7 eV. The spectra of Cp*Rh­(CO)_2_ and CpRh­(CO)_2_ are very similar in shape with maxima at ∼ 4.8 eV and intensities extending toward lower energy transfers. The spectrum of Rh­(acac)­(CO)_2_ exhibits a maximum at the same energy transfer but has a noticeably narrower shape, with a steeper low-energy edge and spectral intensity that extends less toward lower energy transfers. These spectral differences reflect the more closely spaced d-d valence excited states in Rh­(acac)­(CO)_2_ compared to CpRh­(CO)_2_ (and Cp*Rh­(CO)_2_), as previously predicted for the Rh­(acac)­(CO)_2_ to CpRh­(CO)_2_ comparison^41^ and as also observed in the Ir L-edge RIXS spectra of the iridium analogues of the three dicarbonyl complexes studied here.^65^

For a detailed analysis of how CO-to-alkane ligand substitution is reflected in the valence-electronic structures of the three studied Rh complexes, we refer to the RIXS difference spectra shown in Figure 2 (bottom). These difference spectra directly characterize the d-d valence excitations in the σ-complexes. They exhibit broad transient intensity bands in the energy transfer range of ∼1–4 eV, thereby showing a substantial overall shift of approximately 1.5 eV in the inelastic scattering features upon going from the dicarbonyl species to the σ-complexes. The similarity in shape compared to their respective dicarbonyl species suggests a constant shift of all underlying transitions. In analogy to previous metal L-edge RIXS studies of metal complexes with varying ligands, this energy transfer shift can be attributed to the weaker ligand field in the σ-complexes with the C–H ligand compared to CO, resulting in lower transition energies for the d-d excitations. This observation is consistent with a previous time-resolved Fe L-edge RIXS study of Fe­(CO)_5_,^40^ where the photoinduced substitution of a CO ligand by a weaker ethanol ligand was found to reduce the d-d transitions by around 1.5 eV. Similar ligand field changes upon ligand substitution were observed in steady-state metal L-edge RIXS studies for other metal complexes.^66–72^

The experimentally observed differences between the RIXS spectra of the CpRh­(CO)-octane and Rh­(acac)­(CO)-octane σ-complexes are in line with several of our theoretical predictions.^41^ First, we quantitatively confirm by experiment the calculated ligand-field reduction and the corresponding overall shift of d-d valence excitations of around 1.5 eV upon substituting a CO with an alkane ligand in both complexes. Second, we observe a narrower band of d-d valence excitations in Rh­(acac)­(CO)-octane relative to CpRh­(CO)-octane, consistent with the calculated smaller energetic spread of the occupied Rh 4d-derived valence orbitals in Rh­(acac)­(CO)-octane.^41^ Third, our experimental spectra confirm the predicted lower onset in the energy transfer region of 1–2 eV in the Rh­(acac)­(CO)-octane σ-complex, confirming the prediction that the HOMO–LUMO splitting is larger in Rh­(acac)­(CO)-octane than in CpRh­(CO)-octane.^41^

Going back to the transient Rh L_3_-edge X-ray absorption spectra (Figure 2, top), the complete disappearance of the pre-edge peaks in the Cp*Rh­(CO)-octane and CpRh­(CO)-octane σ-complex XAS spectra on nanosecond time scales along with the simultaneous rise of a positive absorption feature at 3006.6 eV (top panels of Figure 2a and b) indicates the progression of the reactions for these two complexes toward C–H activation via oxidative addition (for Rh­(acac)­(CO)_2_, in contrast, the reaction stops with σ-complex formation^49^). This transformation takes place with time constants τ_C–H act_ of 9.5 ± 0.5 ns for Cp*Rh­(CO)_2_ and 14 ± 2 ns for CpRh­(CO)_2_ (corresponding time traces along with a fitted kinetic model can be found in the SI, Figure S6 and Section S2.2). Our finding of a faster C–H activation step by Cp*Rh­(CO)_2_ compared to CpRh­(CO)_2_ is consistent with a previous time-resolved IR spectroscopy study which also reported faster activation in octane by Cp*Rh­(CO)_2_ compared to CpRh­(CO)_2_.^10^ However, while our time constant of 14 ± 2 ns for C–H activation in octane with CpRh­(CO)_2_ agrees with the value reported with IR,^10^ the value we obtain for Cp*Rh­(CO)_2_, 9.5 ± 0.5 ns, is notably shorter than the ∼ 12 ns from IR.^10^ The exact reason for this discrepancy is not clear to us but we speculate that it might be due to the better temporal resolution in our experiment (understanding that we cannot exclude it being due to the different probing mechanisms in XAS and IR spectroscopy). The previous IR study also proposed that the difference between Cp*Rh­(CO)_2_ and CpRh­(CO)_2_ in the time constants of the activation step is due to differences in time scales of octane rearrangements in the σ-complexes that lead to favorable and faster C–H activation with Cp*Rh­(CO)_2_. Our Rh-specific electronic structure probe, in contrast, suggests that differences in metal-alkane bonding within the two σ-complexes dominate the observed differences in activation times. This notion is supported by our earlier findings^20^ that Rh L_3_-edge XAS is not sensitive to different arrangements of octane molecules bound to the Cp*Rh­(CO)-octane and CpRh­(CO)-octane σ-complexes. Instead, as in ref 20 and motivated by the agreement between experiment and theory in the present study, we interpret X-ray spectroscopy as a sensitive probe of variations in the local electronic structure at the Rh center. Due to the + I effect from the five methyl groups on the Cp* ring, we expect the Rh center to be more electron rich for Cp*Rh­(CO)-octane and by extending our previous analyses and observations^20^ we demonstrate in the following how our time-resolved XAS and RIXS observables can probe this.

## Discussion

The measured and calculated Rh L_3_-edge X-ray absorption spectra of the Cp*Rh­(CO)-octane, CpRh­(CO)-octane, and Rh­(acac)­(CO)-octane σ-complexes are shown in Figure 3. To compare intensity differences in detail, the experimental spectra were scaled to match for all three the depletion of the CpRh­(CO)-octane spectrum at 3007.5 eV. This makes the spectral comparison independent of differences in photolysis yields for σ-complex formation (see Methods section). In the calculated spectrum of CpRh­(CO)-octane, the maximum of the pre-edge peak was scaled to match the maximum of the pre-edge peak of the measured spectrum at 10 ps. The same scaling factor was applied to the calculated difference spectra of Cp*Rh­(CO)-octane and Rh­(acac)­(CO)-octane.

**Figure 3:**
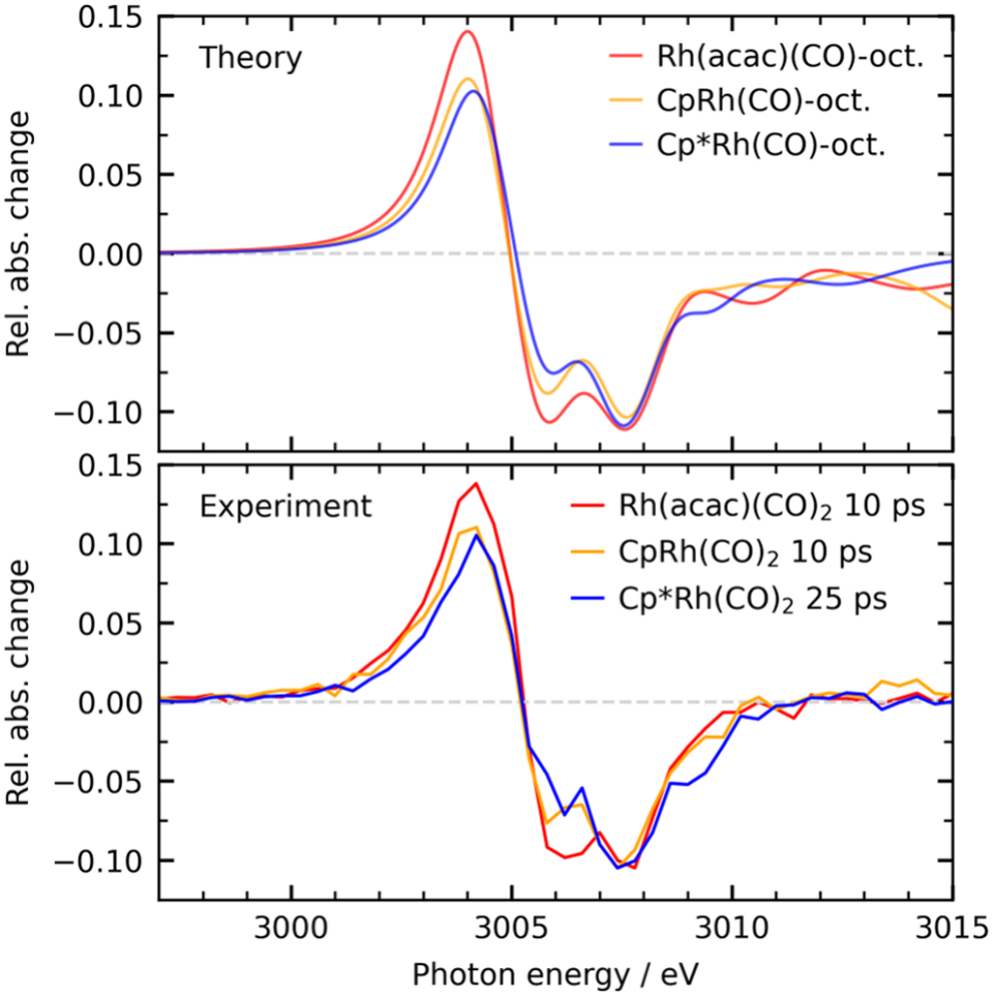
Calculated (top) and measured (bottom) difference XAS spectra of the Cp*Rh­(CO)-octane, CpRh­(CO)-octane and Rh­(acac)­(CO)-octane σ-complexes.

The calculated spectra accurately reproduce the measured ones, capturing the energies, shapes and intensities of both the pre-edge peaks at 3004 eV and the main-edge depletions at 3006 eV. The calculations confirm that the pre-edge peaks in all three systems originate from transitions from the Rh 2p orbital to the lowest unoccupied molecular orbital (LUMO) (see SI Figure S9 for stick spectrum with individual transitions and details of transitions in Tables S2–S4). The LUMO corresponds to the out-of-phase molecular orbital combination formed by σ-donation between the alkane C–H σ-orbital and the empty Rh 4d_*yz*_ orbital (see SI, Figure S11). It thus reflects the accepting orbital in the C–H to Rh donation. For simplicity, it is referred to as LUMO throughout, even though in Rh­(acac)­(CO)-octane it is formally the LUMO+1 due to different orbital energy ordering. Importantly, the LUMO has the same character in all σ-complexes and we can therefore use it to analyze differences in bonding caused by the different ancillary ligands.

The pre-edge peaks in the spectra of the different σ-complexes vary in intensity and not in energy. For Rh­(acac)­(CO)-octane the pre-edge peak is highest and it decreases notably upon substitution of the acac ligand with Cp, as we reported previously.^20^ Here we present a small but significant further decrease in intensity of that pre-edge peak when going to Cp*Rh­(CO)-octane. The decrease of the underlying Rh 2p → LUMO transitions (acac > Cp > Cp*) reflects the decreasing Rh 4d character in the LUMOs (acac > Cp > Cp*) and increasing orbital mixing of the ancillary ligands with the Rh 4d_*yz*_ orbital in the LUMOs (acac < Cp < Cp*). From our calculated σ-complex XAS spectra we extract a decrease of the Rh 4d character from 52.2% in Rh­(acac)­(CO)-octane to 39.3% in CpRh­(CO)-octane and to 35.5% in Cp*Rh­(CO)-octane (see Table 1). Importantly, with decreasing Rh 4d character and concomitantly increasing orbital mixing, the Rh Mulliken charge (positive) decreases from 0.46 in Rh­(acac)­(CO)-octane to 0.37 in CpRh­(CO)-octane and to 0.34 in Cp*Rh­(CO)-octane (Table 1), indicating a corresponding increase in the metal’s electron richness.

**Table 1:** Calculated Rh 4d Character of the LUMO and Mulliken Charge (Positive) in Cp*Rh­(CO)-Octane, CpRh­(CO)-Octane and Rh­(acac)­(CO)-Octane

σ-complex	LUMO Rh 4d character/%	Rh Mulliken charge
Rh(acac)(CO)-octane	52.2	0.46
CpRh(CO)-octane	39.3	0.37
Cp*Rh(CO)-octane	35.5	0.34

These observations establish a correlation between the valence electronic structures of the σ-complexes and their reactivities toward C–H activation: The lower the XAS pre-edge peak, the higher the electron density at the Rh center due to a higher degree of orbital mixing with the ancillary ligands, and the higher the reactivity of the σ-complex toward C–H activation. We deduce the latter correlation because a more stable σ-complex features a larger time constant for the activation step and a lower reactivity for C–H activation. This notion is also based on the approximation that electron density at a metal center is inversely proportional to orbital mixing with its ancillary ligands and proportional to the degree of metal-centered (here Rh 4d) character of the unoccupied orbital involved in orbital interactions with the ancillary ligands. Greater reactivity correlating with higher electron density on the metal center is consistent with established qualitative trends for C–H activation with metals by oxidative addition.^8^ In our case, Rh­(I) in Cp*Rh­(CO)-octane exhibits the highest propensity of the Rh centers to be oxidized (oxidation occurs to Rh­(III) in the activated metal hydride products in our case).^20^ With the Rh 2p → LUMO transitions, as probed in the pre-edge peak intensities, we now provide an experimental observable that correlates with reactivity as expressed in the time constant of the C–H bond activation step and directly reflects the variation in Rh electron density. In addition, we explain the variations in electron density by variations in orbital mixing of the Rh center with the ancillary ligands.

In our previous comparison of reactive CpRh­(CO)-octane and nonreactive Rh­(acac)­(CO)-octane we calculated that Rh­(acac)­(CO)-octane is more stable than CpRh­(CO)-octane by 4.2 kcal/mol.^20^ In addition, we found that the activation step for Rh­(acac)­(CO)-octane is not feasible because the activated product lies 5.1 kcal/mol above Rh­(acac)­(CO)-octane.

For the comparison of Cp*Rh­(CO)-octane and CpRh­(CO)-octane our calculations show that the CpRh­(CO)-octane σ-complex is more stable than Cp*Rh­(CO)-octane by 1.1 kcal/mol (see SI, Figure S17). However, the calculated transition state barriers for the C–H activation step are very similar (5.1 kcal/mol for CpRh­(CO)-octane vs 5.3 kcal/mol for Cp*Rh­(CO)-octane) and therefore cannot explain the observed faster C–H activation step for Cp*Rh­(CO)-octane. This could be due to limitations of the employed theoretical methods. What we can clearly correlate, however, are the different time constants for activation with the different valence electronic structures of the two σ-complexes as probed with Rh L_3_-edge RIXS.

We show in Figure 4 the calculated RIXS spectra of Cp*/CpRh­(CO)_2_ and Cp*/CpRh­(CO)-octane. As for the measured spectra, all calculated RIXS spectra were generated with the incident photon energy centered at the Rh 2p → LUMO pre-edge peak of the σ-complex XAS spectra to make accessible the electronic transitions between the frontier orbitals HOMO, HOMO–1,···HOMO–5 → LUMO (of the six possible transitions, only five are visible in Figure 4 as one has negligible intensity because the occupied orbital involved in this transition is ligand dominated with very weak Rh 4d character and correspondingly weak transition intensity, a detailed description of all transitions and the corresponding MOs are provided in the SI in sections S2.3.4–2.3.6). Comparing the theoretical RIXS spectra of the dicarbonyl species (Figure 4) with the corresponding experimental RIXS spectra (Figure 2, bottom) reveals good agreement in both energetic positions of the inelastic features and overall spectral shapes. In addition, the spectral shifts observed experimentally upon CO to alkane ligand substitution is accurately reproduced in the theoretical RIXS spectra (compare spectra in Figures 2 and 4). This motivates a more detailed analysis of the calculations to elucidate the differences in valence electronic structures of the two σ-complexes Cp*Rh­(CO)-octane and CpRh­(CO)-octane. To this end, we compare the experimental and theoretical difference RIXS spectra of Cp*Rh­(CO)-octane and CpRh­(CO)-octane in Figure 5 (the difference spectra were generated by subtracting the spectrum of the respective dicarbonyl species from that of the σ-complex). The most prominent difference between the experimental spectra is visible on the high-energy sides of the RIXS bands in the energy transfer region around 5 eV where the intensities in Cp*Rh­(CO)-octane are higher than in CpRh­(CO)-octane (Figure 5a). In agreement with experiment, the calculated spectra show the same difference (Figure 5b). In addition, on the low-energy side of the RIXS bands at 2–4 eV we see both in experiment and theory the intensities being lower in Cp*Rh­(CO)-octane compared to CpRh­(CO)-octane. To quantify these differences, we plot in Figure 5c the differences between the RIXS spectra of Cp*Rh­(CO)-octane and CpRh­(CO)-octane for both measured and calculated spectra. Within the uncertainties of the experimental signal-to-noise ratio, we find close agreement of experiment and theory.

**Figure 4:**
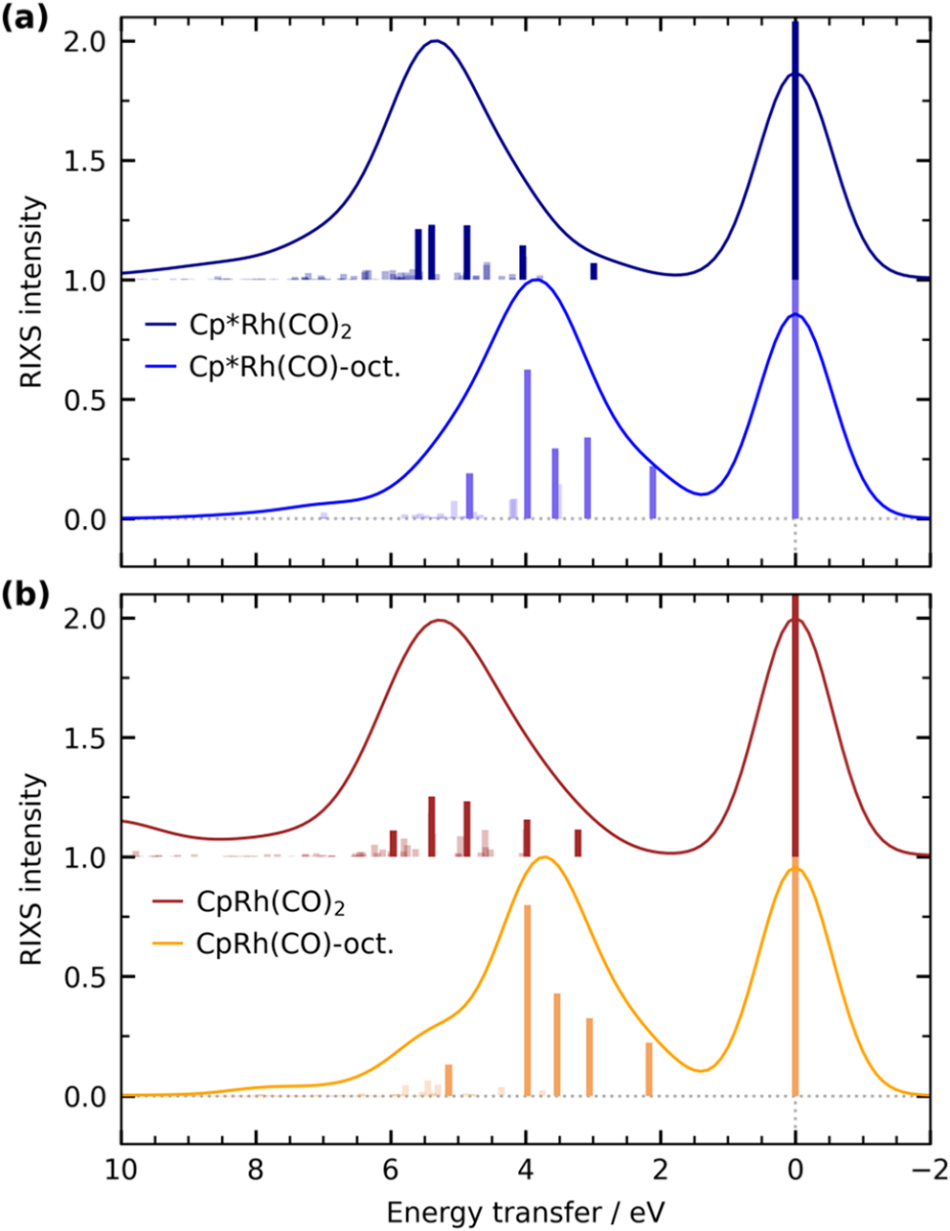
Calculated Rh L_3_ RIXS spectra of (a) Cp*Rh­(CO)_2_ and Cp*Rh­(CO)-octane and (b) CpRh­(CO)_2_ and CpRh­(CO)-octane (calculated for an incident energy of 3004.2 eV matching the pre-edge peak of the σ-complexes, see Figure 3, and normalized to the maximum of their most intense inelastic feature). Individual RIXS transitions are shown as sticks and dominant transitions that determine the shapes of the spectra are highlighted (sticks are scaled using the same scaling factors as for normalization of the line spectra and additionally multiplied in intensity by the same arbitrary scaling factor for better visualization).

**Figure 5:**
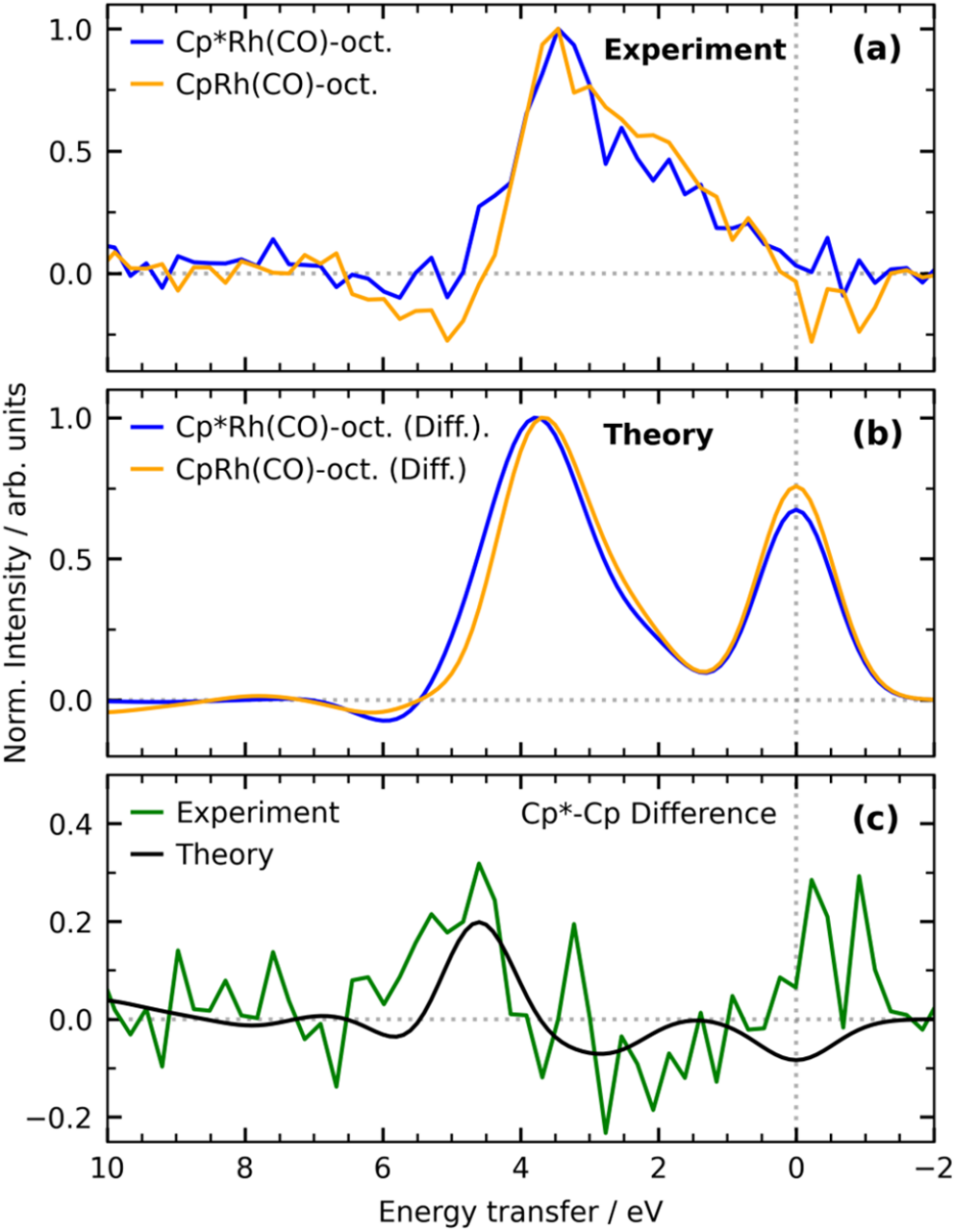
(a) Measured Rh L_3_-edge difference RIXS spectra of Cp*Rh­(CO)-octane and CpRh­(CO)-octane (same data as in Figure 2a,b, bottom, except that the spectrum of CpRh­(CO)-octane was interpolated onto the common energy transfer axis of Cp*Rh­(CO)-octane). (b) Calculated Rh L_3_-edge difference RIXS spectra of Cp*Rh­(CO)-octane and CpRh­(CO)-octane (for the same incidence energy as the experimental spectra). For comparison all spectra are normalized to the intensity of the most intense inelastic feature in each spectrum. (c) Difference of difference spectra (Cp*Rh­(CO)-octane minus CpRh­(CO)-octane for both measured and calculated spectra).

The origin of these differences can be understood by inspecting the individual RIXS transitions assigned to specific electronic transitions between distinct orbitals in the calculated RIXS spectra of Cp*/CpRh­(CO)-octane and Cp*/CpRh­(CO)_2_ (Figure 4, and sections S2.3.4–2.3.6 in the Supporting Information). The higher intensity observed for Cp*Rh­(CO)-octane on the high-energy side of the spectrum (around 5 eV) can be traced back to a difference in energies of the HOMO–5 → LUMO transitions: In Cp*Rh­(CO)-octane, the HOMO–5 → LUMO transition occurs at 4.8 eV and thus 0.3 eV lower in energy compared to CpRh­(CO)-octane where the same transition occurs at 5.1 eV (SI, Tables S5–S8). This difference is evident in the calculated spectra in Figure 4b. In CpRh­(CO)-octane, the HOMO–5 → LUMO transition forms a distinct shoulder at 5.1 eV, somewhat separated from the main band. In Cp*Rh­(CO)-octane, in contrast, the transition is shifted to lower energy and merges with the main band. Consequently, the HOMO–5 → LUMO transition in Cp*Rh­(CO)-octane overlaps less with the main transitions of the Cp*Rh­(CO)_2_ dicarbonyl complex (peaking at around 5.3 eV). In CpRh­(CO)-octane on the other hand, the higher transition energy leads to stronger overlap with the main transitions of the CpRh­(CO)_2_ dicarbonyl complex (also peaking at 5.3 eV). Because the difference spectra are obtained by subtracting the dicarbonyl intensities from those of the σ-complexes, this difference in overlap results in a higher intensity in the difference spectrum of Cp*Rh­(CO)–octane in this energy region.

The more subtle spectral difference on the low-energy transfer side at 2–4 eV can explained by differences in HOMO → LUMO transition energies. This is evident with Figure 4 by comparing the HOMO → LUMO transition energies of the dicarbonyl species with the low-energy sides of the RIXS bands of the σ-complexes: In Cp*Rh­(CO)_2_, the HOMO → LUMO transition (3 eV) is located in the middle of the low-energy side of the RIXS band of Cp*Rh­(CO)-octane. In CpRh­(CO)_2_, in contrast, the corresponding transition (3.2 eV) overlaps less with the low-energy side of the RIXS band of CpRh­(CO)-octane. When subtracting the dicarbonyl intensities from those of the σ-complexes, this results in lower intensity on the low-energy side of the RIXS band (2–4 eV) in the difference spectrum of Cp*Rh­(CO)-octane compared to CpRh­(CO)-octane. The calculated transition energies support this interpretation (SI, Tables S5–S8): Upon CO to C–H ligand substitution, the HOMO → LUMO transition shifts from 3 to 2.1 eV in Cp*Rh­(CO)_2_ (0.9 eV shift) and from 3.2 to 2.2 eV in CpRh­(CO)_2_ (1 eV shift). Figure 4 further shows that the remaining transitions (HOMO–1, HOMO–2, HOMO–3, and HOMO–4 → LUMO) occur at nearly the same energies in both Cp*Rh­(CO)-octane and CpRh­(CO)-octane, as well as in their respective dicarbonyl precursors.

To rationalize the observed differences in the RIXS transitions involving HOMO–5 and HOMO, we first examine the nature of these MOs with the schematic MO-diagram in Figure 6 (note that orbitals are shown for Cp*Rh­(CO)-octane only, orbitals for CpRh­(CO)-octane are nearly identical, see Figure S12 in the SI, and descriptions of molecular orbital interactions with Figure 6 therefore refer to both Cp*Rh­(CO)-octaneand CpRh­(CO)-octane). HOMO and HOMO–5 in Cp*/CpRh­(CO)-octane arise from interactions between the separated Cp*/Cp and Rh^+^(CO)-octane fragments. This decomposition reveals that both MOs originate from the same π-symmetry interaction between the Rh 4d_*yz*_-derived orbitals and the π-orbitals of Cp*/Cp ligand. The lower-lying HOMO–5 corresponds to the in-phase combination of the Cp*/Cp π-orbital with Rh^+^(CO)-octane 4d_*yz*_ orbital and includes additional π*­(CO) admixture. The HOMO, in contrast, arises from the out-of-phase combination of the same Cp*/Cp π-orbital with the Rh^+^(CO)-octane 4d_*yz*_ orbital, now featuring both π*­(CO) and σ*­(C–H) admixtures (a full orbital fragmentation decomposition can be found in the SI, Tables S11 and S12).

**Figure 6:**
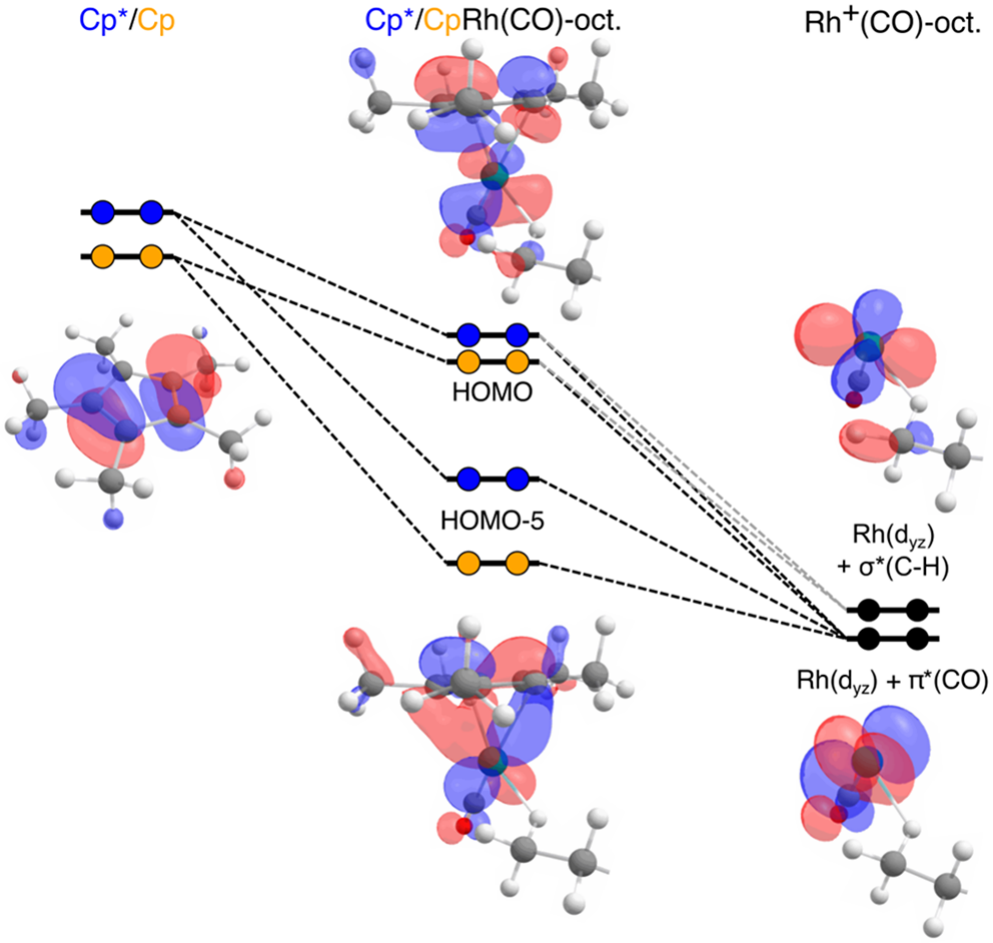
Schematic MO-diagram of Cp*Rh­(CO)-octane and CpRh­(CO)-octane based on the fragments Cp*/Cp and Rh^+^(CO)-octane. The relative orbital energies are as calculated and arranged on an arbitrary energy scale for illustration. The calculated orbitals are all displayed with the same isovalue of 0.043. The HOMO and HOMO–5 are shown for the Cp* system for simplicity. Plots of all MOs and the corresponding RIXS transitions for both systems can be found in the SI, Figure S12.

Importantly, HOMO, by resulting from the out-of-phase combination, cannot contribute to any potential electron delocalization between the Cp*/Cp ligand and the Rh center. HOMO–5, in contrast, by resulting from the in-phase combination, could, in principle, enable such delocalization. Both interacting orbitals, however, the Cp*/Cp π-orbital and the Rh^+^(CO)-octane 4d_*yz*_ orbital, are occupied. Consequently, the interaction resulting in HOMO–5 does not result in net π-bonding but instead gives rise to a repulsive interaction between the Cp*/Cp and Rh^+^(CO)-octane electron densities. This repulsive effect limits delocalization of electron density between the Rh center and the Cp*/Cp ligand and instead favors localization at the Rh center.

With this orbital picture established, the spectral differences observed in both experiment and theory (Figure 5) can be directly correlated with the HOMO–HOMO–5 splitting, rendering the comparison independent of changes in LUMO energies. Based on the calculated RIXS transitions, the HOMO–HOMO–5 splitting amounts to 2.7 eV in Cp*Rh­(CO)-octane and 2.9 eV in CpRh­(CO)-octane (see SI, Tables S5–S8, for exact RIXS transition energies). Because both σ-complexes share the same Rh^+^(CO)-octane fragment, the difference in splitting must originate from the ancillary Cp*/Cp ligand. Consistent with the increased electron density in the Cp* ring introduced by methylating the Cp ring, the π-orbital of Cp* lies higher in energy than that of Cp (Figure 6). This higher energy reduces energetic matching with the Rh 4d_*yz*_-derived orbitals, weakens the π-symmetric interaction, and consequently decreases the splitting between the in-phase (HOMO–5) and out-of-phase combination (HOMO) in Cp*Rh­(CO)-octane relative to CpRh­(CO)-octane. This orbital picture implies that methylation of the Cp ring leads to a decreased delocalization of electron density from the Rh^+^(CO)-octane fragment onto the Cp* ligand (and, correspondingly, an increased electron density at Rh in Cp*Rh­(CO)-octane compared to CpRh­(CO)-octane). To test this, we performed, in a first step, a fragment charge decomposition analysis (Table 2) to quantify charge transfer from the ancillary Cp*/Cp ligands to the Rh^+^(CO)-octane fragment. To then, in a second step, test whether this really affects C–H activation, we also performed another fragment charge decomposition analysis to quantify charge transfer from the Cp*/CpRh­(CO) fragments to the coordinated octane molecule (Table 2).

**Table 2:** Fragment Charge Decomposition Analyses of Cp*Rh­(CO)-Octane and CpRh­(CO)-Octane

	electronic charge transfer (in e^–^)	
complex	Cp*/Cp → Rh^+^(CO)-octane	Cp*/CpRh(CO) → octane
Cp*Rh(CO)-octane	0.82	–0.01
CpRh(CO)-octane	0.72	–0.04

These analyses show that the Cp* ligand donates 0.1 electrons more to Rh^+^(CO)-octane than the Cp ligand. In contrast, the net charge transfer from the Cp*/CpRh­(CO) fragment to the coordinated octane molecule is close to zero in both systems (−0.01 in Cp*Rh­(CO)-octane and −0.04 in CpRh­(CO)-octane), indicating that donation from the C–H σ-orbital to the empty Rh 4d_*xy*_ orbital and back-donation from the Rh 4d_*yz*_ orbital to the C–H σ*-orbital are nearly balanced in both systems (the slightly negative values suggest that donation marginally exceeds back-donation).

Consistent with the fragment charge decomposition analyses, we find in a separate calculation that the Rh center is indeed more electron-rich in Cp*Rh­(CO) (Rh Mulliken charge = 0.31) than in CpRh­(CO) (Rh Mulliken charge = 0.37). The fragment charge analyses further align with differences in the extent of orbital mixing. For the HOMO, Cp*Rh­(CO)-octane exhibits a lower Rh 4d character than CpRh­(CO)-octane (29.3% vs 38.1%, see SI, Tables S9 and S10), indicating stronger mixing with ligand orbitals and enhanced back-donation from the Rh 4d_*yz*_ orbital to the C–H σ*-orbital in the Cp* complex. This increased back-donation is responsible for the enhanced ability of Cp*Rh­(CO)-octane to weaken the C–H bond and is consistent with the experimentally observed faster C–H activation step in Cp*Rh­(CO)-octane.

In contrast, for the HOMO–5, the increased electron density of the Cp* π-orbital reduces delocalization of electron density from the Rh^+^(CO)-octane fragment onto the Cp* ligand, resulting in greater localization and an increased Rh 4d character. Accordingly, HOMO–5 shows a higher Rh 4d character in Cp*Rh­(CO)-octane (47.1%) compared to CpRh­(CO)-octane (41.6%, see SI, Tables S9 and S10), consistent with the larger charge donation from the Cp* ligand observed in the fragment charge decomposition analyses (Table 2). Importantly, the RIXS data thus provide direct experimental evidence that HOMO and HOMO–5 play key roles in regulating electron-density delocalization relevant for C–H activation through π-symmetric interactions between the Cp*/Cp ligands and the Rh^+^(CO)–octane fragment.

We summarize our findings with Figure 7. First, HOMO–5 (Figure 7a, left) reflects a π-symmetric interaction between the occupied Cp*/Cp π-orbital and the occupied Rh 4d_*yz*_ orbital that is repulsive, suppresses electron density delocalization onto the Cp*/Cp ligand and localizes electron density at the Rh center. This effect is stronger in Cp*Rh­(CO)-octane than in CpRh­(CO)-octane due to the higher electron density of the Cp* ligand, yielding a more electron-rich Rh center. Second, classical metal-to-C–H back-donation via the HOMO (Figure 7a, middle) delocalizes electron density from Rh into the C–H σ*-orbital, thereby weakening the C–H bond. The increased electron density localized at Rh enhances this back-donation and promotes oxidative addition during the activation step. Third, donation from the C–H σ-orbital into the empty Rh 4d_*xy*_ orbital (Figure 7a, right) occurs via the LUMO and a more electron-rich Rh center is less susceptible to this interaction. Consequently, donation from C–H to Rh is weaker in Cp*Rh­(CO)–octane than in CpRh­(CO)–octane, which destabilizes the σ-complex and facilitates C–H activation.

**Figure 7:**
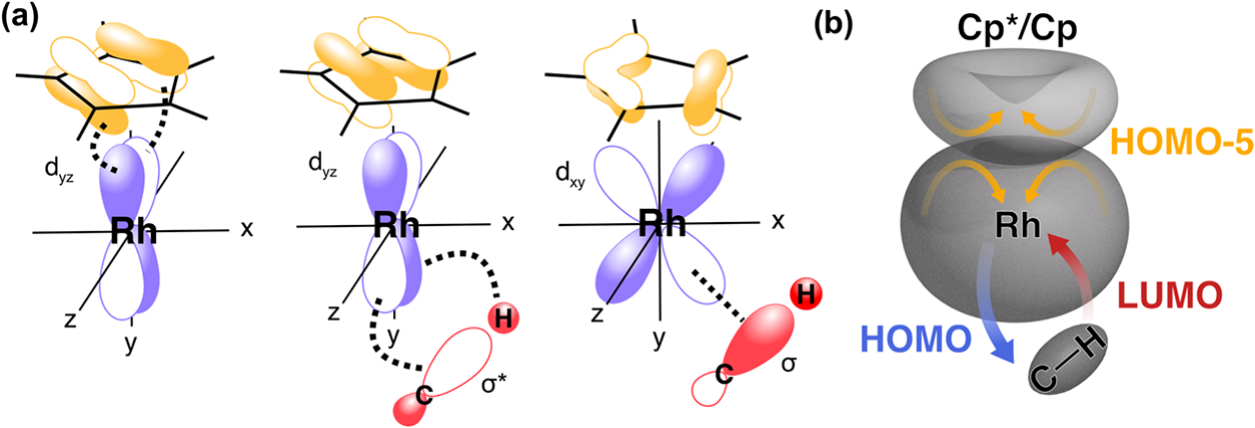
Schematics summarizing (a) the orbital interactions determining (b) electron density regulation and distribution in the studied Cp*/CpRh­(CO)-octane σ-complexes with (a, left) π-symmetry interactions between occupied Cp*/Cp and Rh orbitals via HOMO–5 (a, middle) Rh to C–H back-donation via HOMO, and (a, right) C–H to Rh donation via LUMO. (b) Schematic representation of the combination of all interactions resulting in modulation of electron density at the Rh center.

Together, these three orbital interactions, by alignment of molecular orbital symmetries, regulate the distribution and provision of the necessary electron density at the metal center for efficient donation and back-donation to collectively account for the enhanced reactivity of the Cp* complex by oxidative addition.

These trends, revealed by time-resolved RIXS, further suggest strategies for ligand design. In the presence of the remaining strong π-acceptor CO ligand in the studied σ-complexes, a substantial fraction of the electron density localized at Rh is redistributed toward CO through Rh to CO back-donation, partially compensating the helpful localization by the interaction in the HOMO–5 (Table 2). Replacing CO with a stronger σ-donor and weaker π-acceptor such as trimethylphosphine (PMe_3_) should limit this detrimental redistribution and should help further increase the electron density available for backdonation to the C–H σ*-orbital, thereby enhancing C–H activation. Similar effects have been reported for similar Re carbonyl complexes.^65^

## Conclusion

In summary, we demonstrate the capabilities of time-resolved metal-specific X-ray Absorption Spectroscopy and valence-to-core resonant inelastic X-ray scattering at the metal L_3_-absorption edge with optical pump and X-ray probe experiments for probing the valence electronic structure of transient intermediates in photochemical reactions of metal complexes. We elucidate binding and reactivity toward C–H activation in the photochemically prepared Cp*Rh­(CO)-octane and CpRh­(CO)-octane σ-complexes, the essential intermediates in the related C–H activation reactions. In combination with quantum-chemical calculations of X-ray spectra, molecular orbital interactions and electron densities, we characterize the occupied (using RIXS) and unoccupied (using XAS) valence electronic structures of the short-lived Cp*Rh­(CO)-octane and CpRh­(CO)-octane σ-complexes. Our description of how molecular and atomic orbitals align in symmetry and overlap to interact in these species, enables us to identify the subtle electronic effects that determine stability and reactivity of the studied Rh-alkane σ-complexes. We show, in particular, that time-resolved RIXS, a method uniquely enabled by the intense, tunable and well collimated X-ray beam of an X-ray free-electron laser with X-ray pulse durations in the femtosecond regime, provides direct access to the specific occupied orbitals that facilitate C–H activation by the Rh center.

Our data support and extend established trends in C–H activation, such as the requirement of an electron rich metal center in a low oxidation state.^8^ We provide experimental observables to test fundamental concepts in metal–ligand orbital interactions.^26^ The new information revealed by our approach completes the picture of how bonding regulates reactivity in a metal-alkane σ-complex. We showcase, in particular, the pronounced influence of ancillary ligands (Cp* and Cp in the presented cases) on the electronic structure of the metal center (Rh) by π-symmetry interactions between occupied Cp*/Cp ligand and Rh metal orbitals that result in increasing the “electron richness” at the Rh metal. This highlights the role of ancillary ligands in channeling or tuning electron density to and from the alkane for optimal C–H activation. With X-ray free electron lasers with kHz to MHz repetition rates and corresponding increases in average X-ray flux by orders of magnitude compared to our study (performed at a repetition rate of 100 Hz), time-resolved metal-specific X-ray spectroscopy experiments will enable studying, with the same concepts as discussed here, even smaller electronic-structure effects in even more dilute species as resulting from limited solubility in solution or from minor quantum yields.

## Supplementary Material



## Notes

Optical laser fluence dependency; UV/vis spectra; description of kinetic rate model; delay traces on nanosecond time scale; calculated XAS spectra; list of all calculated RIXS transitions; discussion of RIXS transitions in dicarbonyl complexes and atomic like orbitals in σ-complexes; calculated MOs of dicarbonyl complexes; description of extraction of RIXS spectra; calculated free energy landscapes for activation of all three Rh-complexes; advanced fragment decomposition analysis (PDF)

## #

Max Planck Institute for Chemical Energy Conversion, Stiftstrasse 34–36, D-45470 Mülheim an der Ruhr, Germany

## Abbreviations

NMR: nuclear magnetic resonance
XAS: X-ray absorption spectroscopy
XES: X-ray emission spectroscopy
RIXS: resonant inelastic X-ray scattering
VtC: valence-to-core
XFEL: X-ray free-electron laser
TFY: total fluorescence yield
UV: ultraviolet
FWHM: full width at half-maximum
TD-DFT: time-dependent density functional theory
