# Supplementary material for: Time-Resolved Resonant Inelastic X‑ray Scattering Reveals How Orbital Symmetry Alignment Enables C–H Activation
Source: J Am Chem Soc. 2026 Apr 30;148(18):19423–35. doi: 10.1021/jacs.6c05747 (PMC13185092; doi:10.1021/jacs.6c05747)
Supplement: Supplementary file 1 [file ja6c05747_si_001.pdf]

Supporting Information for Time-resolved Resonant Inelastic X-ray Scattering reveals how Orbital Symmetry Alignment Enables C–H Activation

Timo Dederichs,[*,†] Ambar Banerjee,[*,‡] Victoria Kabanova,[†] Robert Stefanuik,[†] Antonia Freibert,[¶] Emma V. Beale,[§] Florian Dworkowski,[§] Rebeca G. Castillo,[‖,⊥] Philip J.M. Johnson,[§] Claudio Cirelli,[§] Nils Huse,[¶] Camila Bacellar,[§] Raphael M. Jay,[*,†] and Philippe Wernet[*,†]

†*Department of Physics and Astronomy, Uppsala University, 75120 Uppsala, Sweden*

‡*Research Institute for Sustainable Energy (RISE), TCG Centres for Research and Education in Science and Technology (TCG-CREST), Kolkata, 700091 India*

¶*Department of Physics, University of Hamburg, 22761 Hamburg, Germany*

§*Paul-Scherrer Institute, CH-5232 Villigen PSI, Switzerland*

‖*Laboratory of Ultrafast Spectroscopy, Ecole Polytechnique Federale de Lausanne (EPFL), CH-1015 Lausanne, Switzerland*

⊥*Current address: Max Planck Institute for Chemical Energy Conversion, Stiftstrasse 34-36, D-45470 Mülheim an der Ruhr, Germany*

E-mail: timo.dederichs@physics.uu.se; ambar.banerjee@tcgcrest.org; raphael.jay@physics.uu.se; philippe.wernet@physics.uu.se

S1 Methods

S1.1 Fluence dependency

The laser fluence dependency of the pump-probe signal measured at an incidence energy of 3002.8 eV at a pump-probe delay of 250 fs of $Cp^{*}Rh(CO)_2$ in octane solution is shown in Figure S1. The laser fluence behaves linearly below 40 mJ cm^{-2}. the measurements in the main text were done at a fluence of 16.5 mJ cm^{-2} for the XAS and 36.8 mJ cm^{-2} for the RIXS measurements.

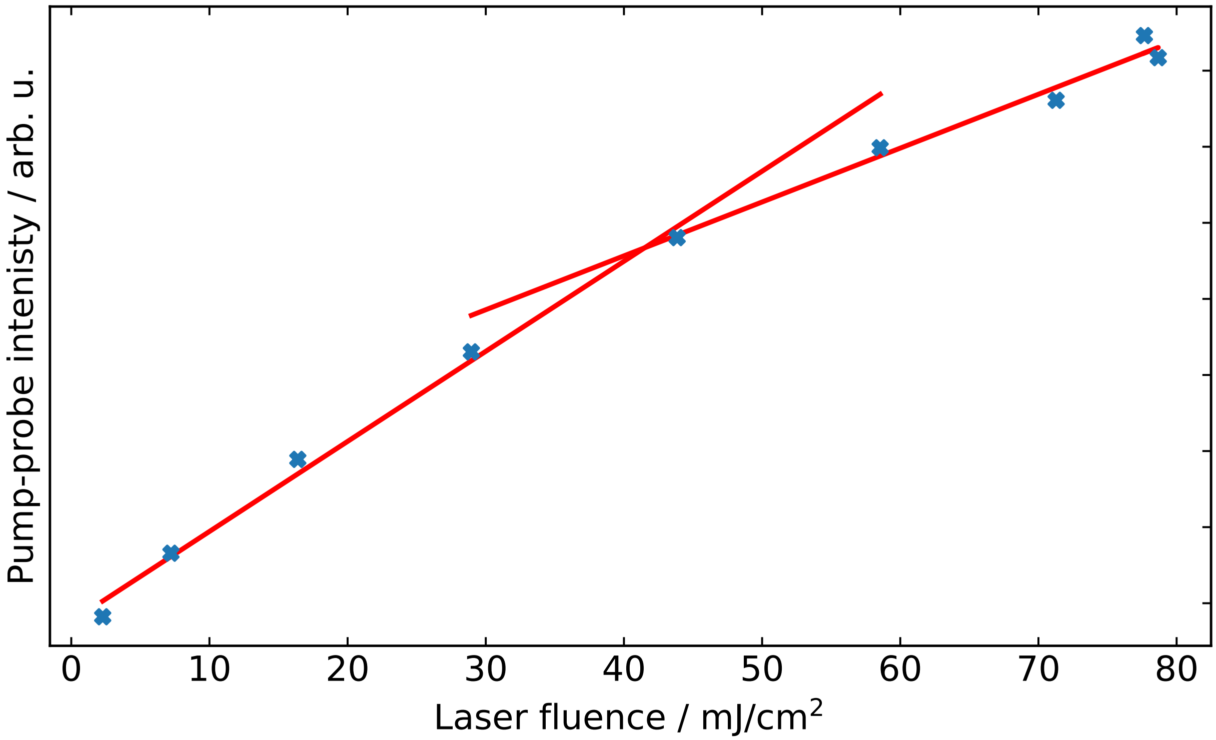

Figure S1: Fluence scan measured at SwissFEL at an incidence energy of 3002.8 eV at a pump-probe delay of 250 fs of $Cp^{*}Rh(CO)_2$ in octane solution.

S1.2 UV-Vis

To assess potential sample degradation induced by exposure to oxygen, UV laser irradiation, and X-rays, UV/Vis spectra were recorded. The spectral profile of a freshly prepared sample batch of Cp*Rh(CO)$_2$ in octane solution was compared with a batch exposed to oxygen for 4 hours and to a batch exposed to X-rays and UV laser irradiation for 3 hours (see Figure S2). All spectra were normalized to the absorption maximum at 239 nm. The spectra exhibit identical spectral features, confirming the sample integrity under the experimental conditions of the measurements reported in the main text and over extended exposure times.

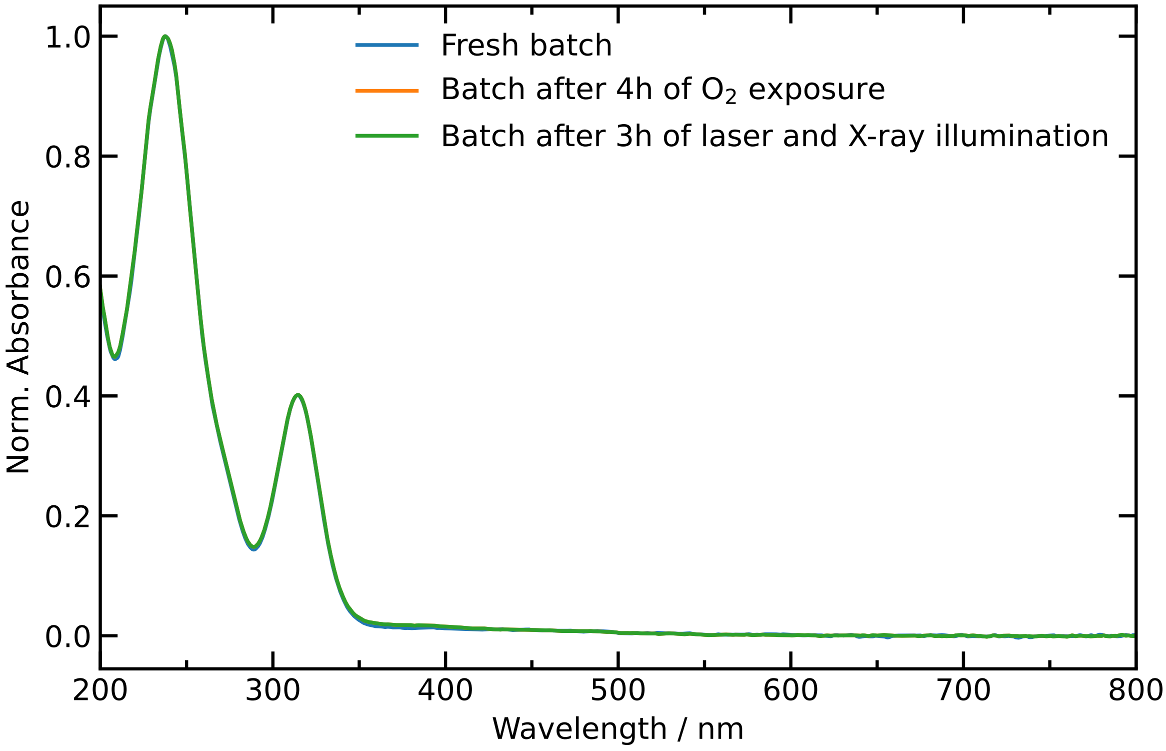

Figure S2: UV-Vis spectra of Cp*Rh(CO)$_2$ in octane solution recorded directly after sample preparation, 4h of O_2 exposure, and after 3h of running the solution in the time-resolved XAS experiment.

S1.3 Heating by pump laser

The temperature rise ΔT induced by irradiation by the UV pump laser is given by the ratio of the absorbed energy E_{abs} and the specific heat capacity C_{p} (at constant pressure) times the irradiated mass m of the sample:

$$\Delta T = \frac{E_{\text{abs}}}{C_{\text{p}} m} \tag{S1}$$

The mass can be written as the product of the density ρ of the sample and the heated volume V_{heated}. Here, to first order, the density ρ of the solution is mainly given by the density of the solvent $\rho_{\text{oct.}}$ (in this case octane). Using this relationship, we can rewrite eq. S1 in the following form:

$$\Delta T = \frac{E_{\text{abs}}}{C_{\text{p}} \rho V_{\text{heated}}} \tag{S2}$$

The energy E_{abs} absorbed by the sample can be calculated from the laser pulse energy E_0 and the fraction of the energy absorbed by the solute at the particular wavelength f_{abs}:

$$E_{\text{abs}} = E_0 f_{\text{abs}} = E_0 (1 - 10^{-A_{266}}) \tag{S3}$$

As the solvent is transparent at the pump wavelength, we assume that the only absorption is due to the solute, Cp*Rh$(CO)_2$, absorbing at this wavelength. The absorbance A_{266} of the solute at the given pump wavelength (here 266 nm) is given by Lambert Beer's law:

$$A_{266} = c d \epsilon_{266}, \tag{S4}$$

where c is the concentration of the solute, d is the thickness, and ϵ_{266} is the molar extinction coefficient of the solute at 266 nm. Based on previous measurements of CpRh$(CO)_2$,[1] we estimate the molar extinction coefficient of Cp*Rh$(CO)_2$ to be ≈ 4500 M^{-1} cm^{-1}.

The second quantity required in eq. S3 is the laser pulse energy E_0, which is given by the laser fluence F and the laser spot size A_{spot}:

$$E_0 = F A_{\text{spot}} \tag{S5}$$

Using eq. S3-S5, and rewriting the heated volume V_{heated} as the product of the laser spot size A_{spot} and the path length (thickness) of the jet d, we can rewrite eq. S2 into the following form:

$$\Delta T = \frac{F(1 - 10^{-A_{266}})}{C_{\text{p}} \rho d} \tag{S6}$$

Using a laser fluence F of $16.5\,\text{mJ}\,\text{cm}^{-2}$, a concentration c of 20 mM, a molar extinction coefficient ϵ_{266} of 4500 M^{-1} cm^{-1}, a path length d of $75\,\mu\text{m}$, the heat capacity C_{p} for octane of $2.24\,\text{J}\,\text{K}^{-1}\,\text{g}^{-1}$,[2] and the density ρ of octance of $0.703\,\text{g}\,\text{cm}^{-3}$[3] results in a UV pump laser induced temperature rise ΔT of 1.10 K. Temperature-dependent X-ray pump-probe spectra of metal complexes in solution have shown changes for temperature differences of 20 K and resulted in intensity changes in the low percent range.[4] We therefore conclude that the observed transient XAS signals at 25 ps do not originate from a pump-laser induced rise of the sample temperature. To further confirm this and based on the assumption that a temperature rise would cause a transient shift of the XAS spectrum, we computed the first derivative of the steady-state XAS spectrum of Cp*Rh$(CO)_2$ shown together with the steady-state spectrum and the corresponding transient 25 ps spectrum in Figure S3. Comparison of the derivative and the 25 ps spectrum reveals no match in energy, but a significant larger XAS difference signal at 25 ps (as expected from a change in bonding and valence electronic structure), confirming that it does not originate from a UV pump induced temperature rise.

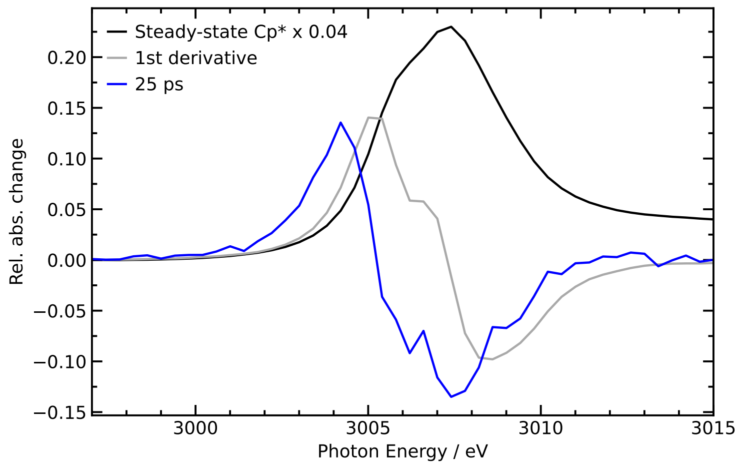

Figure S3: Steady-state Rh L3-edge X-ray absorption spectrum of Cp*Rh$(CO)_2$ along with its first derivative and the transient 25 ps spectrum.

S2 Results and discussion

S2.1 Picosecond delay traces

S2.1.1 Experimental delay traces

Figure S4 shows the delay traces of the three Rh complexes Cp*Rh$(CO)_2$, CpRh$(CO)_2$ and Rh(acac)$(CO)_2$ in octane solution measured at a photon energy of 3002.8 eV.

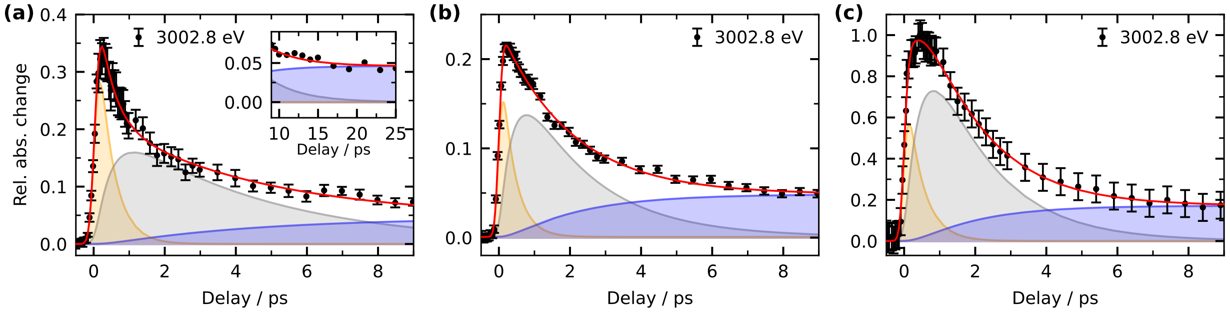

Figure S4: Delay traces of (a) Cp*Rh$(CO)_2$, (b) CpRh$(CO)_2$ and (c) Rh(acac)$(CO)_2$ (relative absorption changes versus pump-probe delays measured at 3002.8 eV as data points with error bars) and results of fits of a kinetic model to the data (solid lines and shaded areas). The orange, gray, and blue shaded areas are assigned to the relative populations of excited species, the monocarbonyl fragments, and the octane σ-complexes, respectively. The red curves illustrate biexponential fits of the data ($R^2 = 0.992$ for Cp*Rh$(CO)_2$ and $R^2 = 0.995$ for Rh(acac)$(CO)_2$; the delay trace and fit of CpRh$(CO)_2$ are reproduced from our previous publication[1]).

The experimental data are well described by a kinetic model with three species and a bi-exponential decay to intermediate species that are stable on the time scales of 10 ps for $CpRh(CO)_2$ and $Rh(acac)(CO)_2$ and 25 ps for $Cp^*Rh(CO)_2$. The fitted time constants are shown in Table S1 (see section S2.1.2 for a detailed description of the kinetic model). The time constants are the inverse of the rate constants in the kinetic model. Guided by our previous study of $CpRh(CO)_2$,[1] we assign the fastest time constants $\tau_{CO-Diss.}$ of (420 ± 120) fs for $Cp^*Rh(CO)_2$, (370 ± 50) fs for $CpRh(CO)_2$, and (450 ± 120) fs for $Rh(acac)(CO)_2$ to relaxation of the electronic excited states of the dicarbonyls and concomitant CO dissociation. Within the uncertainties of the measurements, the time constants $\tau_{CO-Diss.}$ are the same for all three complexes.

The intermediate time constants $\tau_{\sigma-complex}$ of (4.3 ± 0.4) ps for $Cp^*Rh(CO)_2$, (2.0 ± 0.1) ps for $CpRh(CO)_2$, and (1.7 ± 0.5) ps for $Rh(acac)(CO)_2$ correspond to the timescales of σ-complex formation, in which an octane molecule binds to the respective free monocarbonyl fragments. As in our previous study,[1] this assignment is based on the observation that for all three complexes there are no new detectable components in the delay traces beyond 10 ps or 25 ps. This observation indicates the formation of a kinetically stable intermediate in all three cases on these time scales. This assignment is confirmed by the calculated X-ray absorption spectra in Figure 3. The time constants for σ-complex formation differ slightly for the three complexes with $Cp^*Rh(CO)_2$ being slower than $Rh(acac)(CO)_2$ and $CpRh(CO)_2$. A possible explanation, which we do not further investigate here, could be steric hindrance introduced in $Cp^*Rh(CO)_2$ by the bulky methyl groups at the Cp* cyclopentadienyl ring which may hinder the octane molecule to coordinate to the free coordination site of the Cp*RhCO monocarbonyl fragment.

Table S1: Time constants of the kinetic-model fits of the delay traces of $Cp^*Rh(CO)_2$, $CpRh(CO)_2$ and $Rh(acac)(CO)_2$. The time constants are the inverse of the rate constants k_1, k_2 and k_3 mentioned in section S2.2.

	$\tau_{CO-Diss.}$ / fs	$\tau_{\sigma-complex}$ / ps	$\tau_{C-H\ act.}$ / ns
$Cp^*Rh(CO)_2$	420 ± 120	4.3 ± 0.4	9.5 ± 0.5
$CpRh(CO)_2$	370 ± 50	2.0 ± 0.1	14 ± 2
$Rh(acac)(CO)_2$	450 ± 120	1.7 ± 0.5	/

S2.1.2 Kinetic Rate Model

The kinetic rate model used to describe the femtosecond dynamics following 266 nm laser pulse excitation of the three complexes of $Cp^*Rh(CO)_2$ consists of three transient species and is analogous to that employed in a previous publication.[1,5] Upon photoexcitation, we propose (detailed for $Cp^*Rh(CO)_2$ and analogous for the other two complexes) that these species evolve sequentially from the electronically excited state of the intact $Cp^*Rh(CO)_2$ complex (MLCT) via the free Cp*Rh(CO) monocarbonyl fragment (FF) to the alkane-bound σ-complex Cp*Rh(CO)-octane (σ-alk). The latter is meta-stable on a picosecond (ps) timescale and subsequently transforms to the C-H bond-activated product (C-H) on nanosecond timescales.

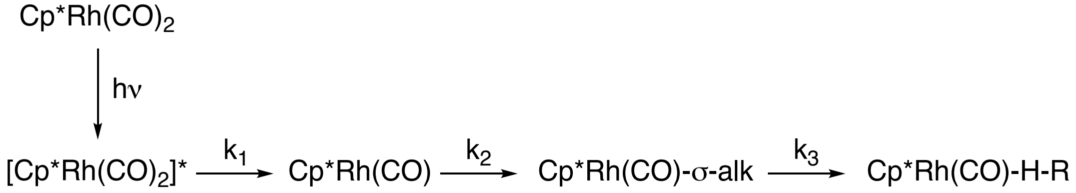

Figure S5: Reaction scheme of the photoinduced C-H bond activation used for the three complexes studied here and detailed with $Cp^*Rh(CO)_2$ with the corresponding rate constants k_1, k_2, k_3. For $Rh(acac)(CO)_2$ the last activation step does not occur.

To obtain the overall rate equation for this reaction, we start from the differential rate

equation for each species.

$$\frac{\mathrm{d[MLCT]}}{\mathrm{d}t} = -k_1[\mathrm{MLCT}] \tag{S7}$$

$$\frac{\mathrm{d[FF]}}{\mathrm{d}t} = k_1[\mathrm{MLCT}] - k_2[\mathrm{FF}] \tag{S8}$$

$$\frac{\mathrm{d}[\sigma\mathrm{-alk}]}{\mathrm{d}t} = k_2[\mathrm{FF}] - k_3[\sigma\mathrm{-alk}] \tag{S9}$$

$$\frac{\mathrm{d[C-H]}}{\mathrm{d}t} = k_3[\sigma\mathrm{-alk}] \tag{S10}$$

Integrating the differential equations yields the time-dependent populations for these species:

$$[\mathrm{MLCT}] = [\mathrm{MLCT}]_0 \exp(-k_1 t) \tag{S11}$$

$$[\mathrm{FF}] = [\mathrm{MLCT}]_0 \frac{k_1}{k_2 - k_1} \left(\exp(-k_1 t) - \exp(-k_2 t)\right) \tag{S12}$$

$$\begin{aligned}[\sigma\mathrm{-alk}] &= [\mathrm{MLCT}]_0 k_1 k_2 \frac{k_3\left(\exp(-k_1 t) - \exp(-k_2 t)\right) + k_2\left(\exp(-k_3 t) - \exp(-k_1 t)\right)}{(k_2 - k_1)(k_3 - k_2)(k_3 - k_1)} \\ &+ [\mathrm{MLCT}]_0 k_1 k_2 \frac{k_1\left(\exp(-k_2 t) - \exp(-k_3 t)\right)}{(k_2 - k_1)(k_3 - k_2)(k_3 - k_1)} \end{aligned} \tag{S13}$$

$$[\mathrm{C-H}] = [\mathrm{MLCT}]_0 - [\mathrm{MLCT}] - [\mathrm{FF}] - [\sigma\mathrm{-alk}] \tag{S14}$$

As the σ-complexes decay on nanosecond timescale, the time constants for this step are substantially slower compared to the previous step ($k_3 <<< k_2$), which allows to set the time constant for activation step approximately to zero ($k_3 \approx 0$; as a result the concentration of the activated species is also zero $[\mathrm{C-H}] \approx 0$. For $\mathrm{Rh(acac)(CO)_2}$ the reaction does not proceed towards C–H activation and thus the rate constant is exactly 0). Thus, equation S14 can be neglected to a first approximation. Due to the significant spectral broadening (see transient Rh $\mathrm{L_3}$-edge X-ray absorption spectra in the main manuscript), it is justified to assume that all three remaining species contribute (to a varying degree) to the measured intensity at the pre-edge. Additionally, the concentration of the initially excited species $[\mathrm{MLCT}]_0$ is unknown. Therefore, the concentrations are replaced with individual scaling

factors for each species to account for both these aspects. This results in a new set of rate equations:

$$[\text{MLCT}] = \exp(-k_1 t) \tag{S15}$$

$$[\text{FF}] = \frac{k_1}{k_2 - k_1} \left(\exp(-k_1 t) - \exp(-k_2 t)\right) \tag{S16}$$

$$[\sigma{-}\text{alk}] = 1 - \frac{k_2 \exp(-k_1 t) - k_1 \exp(-k_2 t)}{k_2 - k_1} \tag{S17}$$

The sum of these three equations describes the time-dependent intensity change in the pre-edge:

$$I_{3002.8\text{eV}} = a[\text{MLCT}] + b[\text{FF}] + c[\sigma{-}\text{alk}], \tag{S18}$$

with a, b and c being individual scaling factors. To fit the experimental data with this kinetic model, each exponential term in the rate equations is convolved with a Gaussian representing the instrument response function, as well as with an error function describing the nearly instantaneous rise at time zero. This yields the following expression for the modified exponentials:

$$\exp_{\text{conv.}}(t) = \frac{1}{2} \exp\left(\frac{k\left(2\mu + k\sigma^2 - 2t\right)}{2}\right) \text{erfc}\left(\frac{\mu + k * \sigma^2 - x}{\sqrt{2}\sigma}\right) \tag{S19}$$

Here, μ denotes time-zero, and σ the temporal width of the instrument response which will be discussed in greater detail in section S2.1.3.

S2.1.3 Temporal resolution and instrument response

The instrument response function (IRF) was determined to characterize the temporal resolution of the experiment. Fitting the picosecond delay traces shown in Figure S4 of $Cp^*Rh(CO)_2$ yields an temporal width σ of 114 fs, which corresponds to a $FWHM_{IRF}$ of 270 fs. This temporal resolution arises from several independent contributions (all values reported as FWHM). The most prominent factors are the durations of the UV pump pulse ($\approx$ 75 fs) and the X-ray probe pulse ($\approx$ 40 fs). Because the X-ray pulses are generated statistically via the SASE process, their arrival time exhibits an intrinsic timing jitter ($\approx$ 60 fs). In addition, the group velocity mismatch (GVM) between the UV and X-ray pulses introduces further temporal broadening, estimated to be approximately 1 fs per µm of jet thickness, corresponding to $\approx$ 75 fs under the present experimental conditions. Finally, slow drifts of the temporal overlap (time zero), caused by changes in the optical path of the UV laser, contributes additional broadening. Typically this can be accounted for by using a time-tool provided at the cost of reduced flux in the tender regime. Additionally, deposition of the volatile solvent on the coupling optics can lead to timing shift of several hundreds femtoseconds per hour. Although each individual run was corrected for this drift, residual fluctuations during acquisition remain. We estimate that this effect contributes approximately 200 fs to the overall temporal broadening. Based on these aspects, we can calculate the theoretical instrument response function according to equation S20:

$$\mathrm{FWHM_{IRF}} = \sqrt{\mathrm{FWHM}^2_{\mathrm{pump}} + \mathrm{FWHM}^2_{\mathrm{probe}} + \mathrm{FWHM}^2_{\mathrm{jitter}} + \mathrm{FWHM}^2_{\mathrm{GVM}} + \mathrm{FWHM}^2_{\mathrm{drift}}} \tag{S20}$$

Plugging in the values listed above yields a theoretical value of 238 fs for $FWHM_{IRF}$. This small discrepancy of 30 fs between this estimate and the value obtained from the fit likely arises from uncertainties in the individual contributions, as well as from the fact that some effects (e.g., timing jitter and group velocity mismatch) are not strictly Gaussian and are

therefore only approximately represented by Gaussian FWHM terms.

S2.2 Nanosecond delay traces of Cp*Rh(CO)$_2$

Figure S6 shows the nanosecond delay traces of Cp*Rh(CO)$_2$ measured at incidence energies of 3004 eV and 3006.6 eV. These incident energies were chosen to monitor the decay of the pre-edge feature associated with the octane σ-complex (3004 eV) and the recovery of the ground state bleach (3006.6 eV). The data sets were globally fitted using a monoexponential decay model, resulting in a time constant of 9.5 ± 0.5 ns.

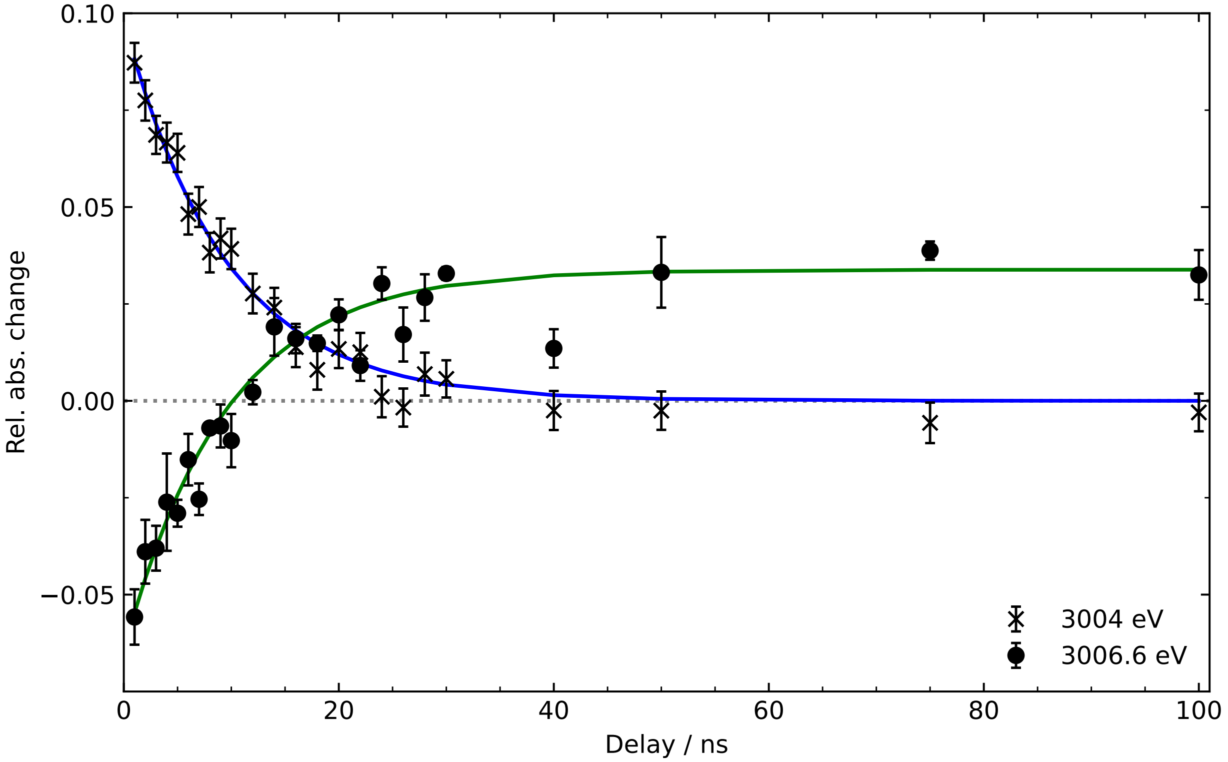

Figure S6: Delay traces of Cp*Rh(CO)$_2$ measured at an photon energy of 3004 eV (cross) and 3006.6 eV (dot) with error bars. The result of the global fit is shown in colored lines.

S2.3 Theoretical calculations

S2.3.1 Calculated XAS spectra

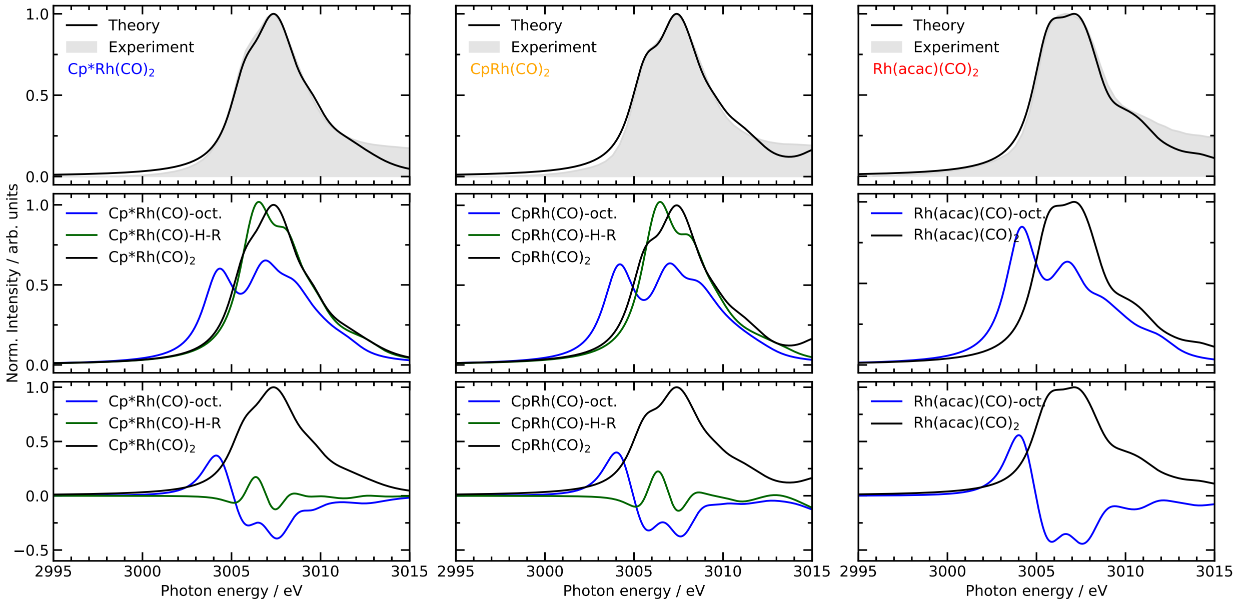

Figure S7: (Top) Experimental steady-state Rh L_3-edge XAS spectrum (shaded) of Cp*Rh$(CO)_2$ (left), CpRh$(CO)_2$ (middle) and Rh(acac)$(CO)_2$ (right) compared to their calculated versions (black line), respectively. In all cases, the experimental and calculated spectra are normalized to their maximum intensity. All calculated spectra were shifted by $-22.25\,$eV to account for the neglected core-hole relaxation in the calculation. (Middle) Calculated spectra of the dicarbonyl, σ-complex and C-H activated product of Cp*Rh$(CO)_2$ (left), CpRh$(CO)_2$ (middle) and Rh(acac)$(CO)_2$ (right), respectively. The respective difference spectra are shown in the panels below.

Figure S7 compares the experimental steady-state Rh L_3-edge XAS spectra of Cp*Rh$(CO)_2$, CpRh$(CO)_2$ and Rh(acac)$(CO)_2$ with their corresponding calculated spectra. All calculated spectra were shifted by $-22.25\,$eV to account for the core-hole relaxation, which is neglected in the calculations. This shift was determined by calculating the maximum of the cross-correlation between the respective experimental and calculated spectra. For further validation of the spectral assignment of the σ-complexes, the transient XAS spectra of the three Rh complexes at 10 ps (or at 25 ps in the case of Cp*Rh$(CO)_2$) are overlaid with the corresponding calculated spectra of the respective σ-complexes (see Figure S8).

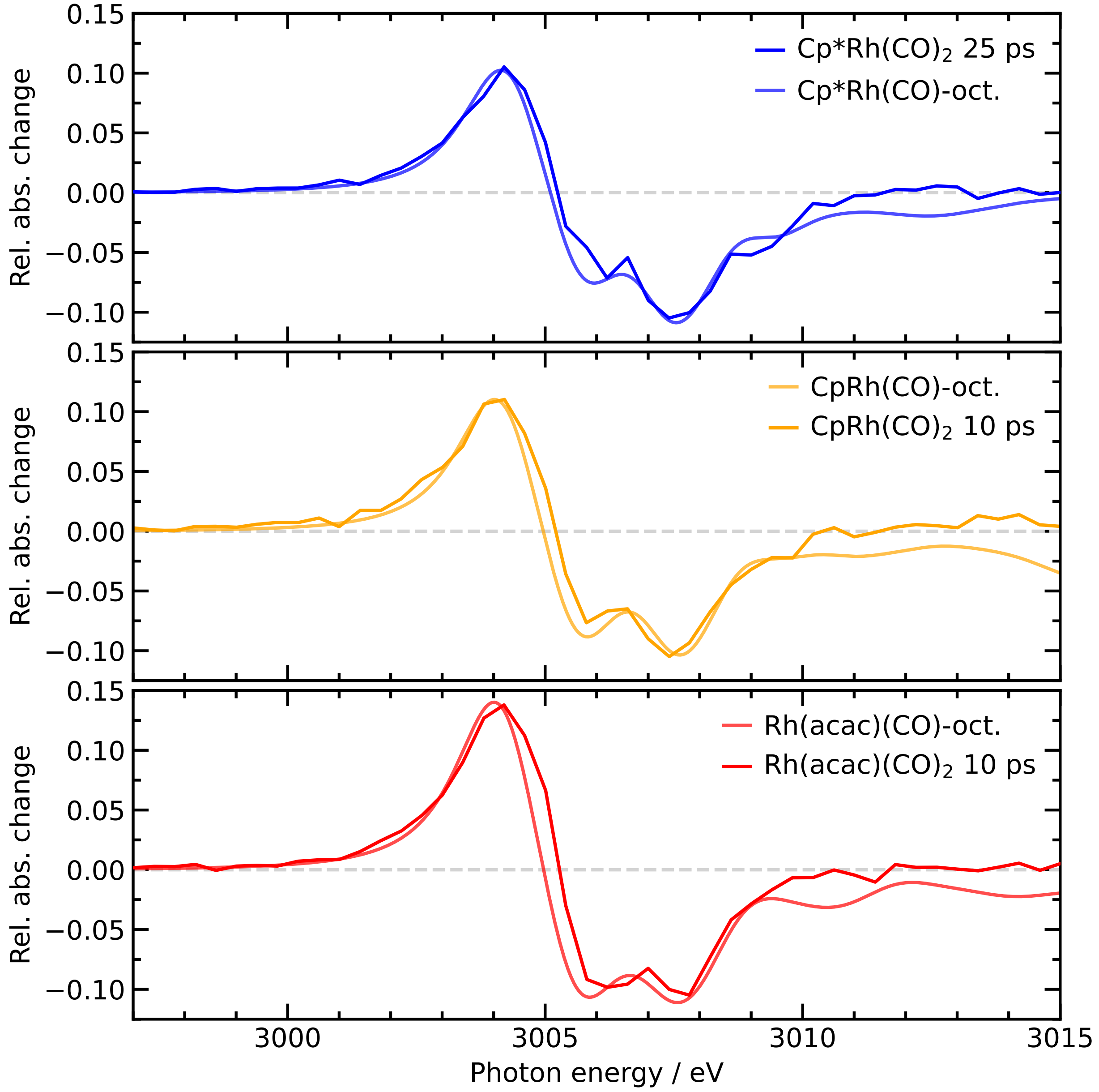

Figure S8: Comparison of experimental and theoretical difference Rh L_3-edge XAS spectrum of the σ-complexes. (Top) comparison of Cp*Rh(CO)-octane, (middle) CpRh(CO)-octane and (bottom) Rh(acac)(CO)-octane.

The transitions underlying the XAS spectra of both dicaronly species and the corresponding σ-complexes are shown in Figure S9.

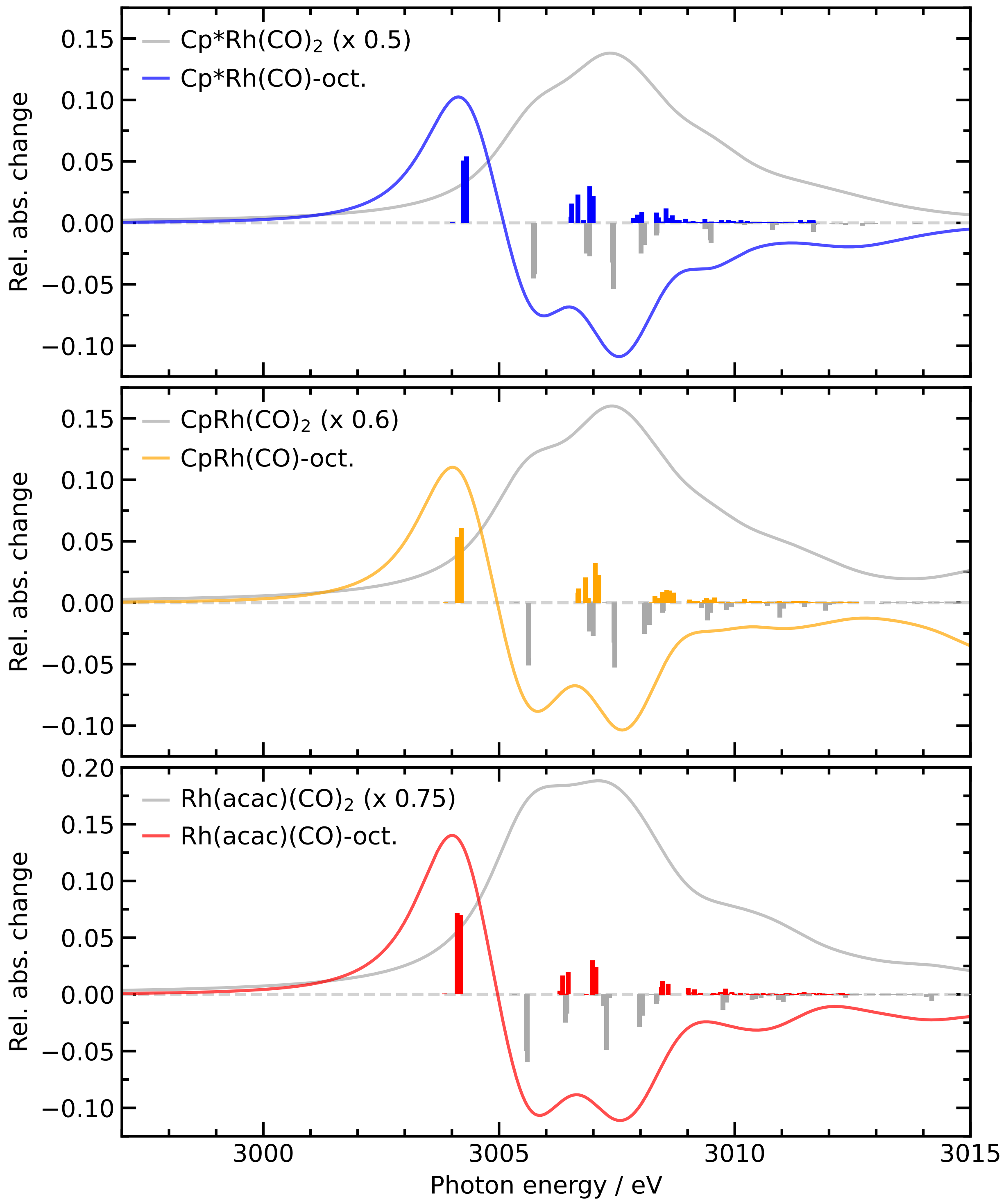

Figure S9: Theoretical Rh L_3-edge XAS spectra of (top) Cp*Rh$(CO)_2$, (middle) CpRh$(CO)_2$, and (bottom) Rh(acac)$(CO)_2$, together with the corresponding difference spectra of the σ-complexes and their respective stick spectra.

In all cases, the pre-edge in the difference spectra of the σ-complexes arises from three intense transitions, where two are similar in energy and thence two sticks are visible in Figure S9. For each transition, the corresponding orbital contributions and their relative weights are reported in Tables S2 to S4. This analysis reveals that the pre-edge features originate almost exclusively from excitations from the three Rh 2p orbitals (orbital no. 2-4) to the LUMO, allowing for a one-electron picture interpretation in XAS and RIXS.

Table S2: Rh L_3-edge XAS transitions for Cp*Rh(CO)-octane. Contributions with less than 1% were not listed. Orbital no. 100 corresponds to the LUMO.

Transition	Energy / eV	Initial orbital	Final orbital	Contribution / %
1	3004.0	4	100	93.7
		4	101	1.1
2	3004.2	3	100	93.6
		3	101	1.0
3	3004.3	2	100	93.6

Table S3: Rh L_3-edge XAS transitions for CpRh(CO)-octane. Contributions with less than 1% were not listed. Orbital no. 80 corresponds to the LUMO.

Transition	Energy / eV	Initial orbital	Final orbital	Contribution / %
1	3003.9	4	80	95.0
		4	81	1.1
2	3004.2	3	80	94.8
3	3004.3	2	80	94.8

Table S4: Rh L_3-edge XAS transitions for Rh(acac)(CO)-octane. Contributions with less than 1% were not listed. Orbital no. 90 corresponds to the LUMO+1, however this has the same character as the LUMO of the Cp* and Cp system and thus referred as LUMO in the manuscript.

Transition	Energy / eV	Initial orbital	Final orbital	Contribution / %
1	3003.8	4	90	94.0
2	3004.1	3	90	93.7
3	3004.2	2	90	94.1

S2.3.2 Calculated RIXS transitions

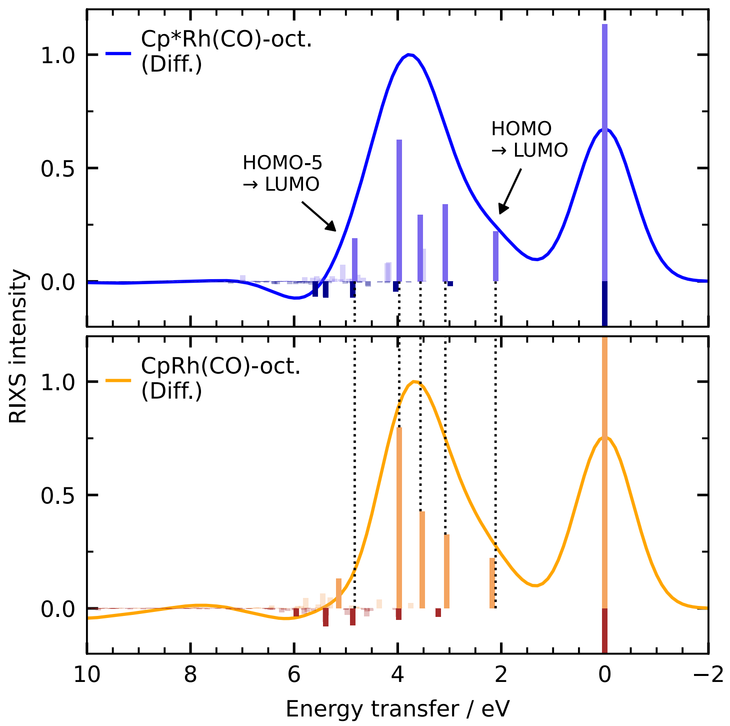

Figure S10: Calculated Rh L_3 difference RIXS spectra of Cp*Rh(CO)-octane and CpRh(CO)-octane. Individual RIXS transitions are shown as sticks and dominant transitions that determine the shapes of the spectra are highlighted. All spectra are normalized to the maximum of their most intense inelastic feature. Sticks are scaled using the same scaling factors as for normalization of the line spectra and additionally multiplied in intensity by the same arbitrary scaling factor for better visualization. Sticks pointing up in the difference spectra originate from RIXS intensities due to the respective σ-complex while sticks pointing down originate from bleached transitions of the respective dicarbonyl species.

Figure S10 shows the calculated difference RIXS spectra of Cp*/CpRh$(CO)_2$ and Cp*/CpRh(CO)-octane along with the underlying transitions (shown as vertical sticks). As for the experimental spectra, all calculated RIXS spectra were generated with the incident photon energy being centered at the LUMO resonance of the σ-complexes.

Tables S5 to S8, show the most dominant RIXS transition for Cp*Rh$(CO)_2$, Cp*Rh(CO)-octane, CpRh$(CO)_2$ and CpRh(CO)-octane. For each transition, the initial and final state (number of orbitals included in the transition) are given along with a description of which molecular orbitals are participating in the respective species. For each species, five (instead

of the expected six) transitions HOMO $\rightarrow$ LUMO, HOMO-1 $\rightarrow$ LUMO,... HOMO-5 $\rightarrow$ LUMO are shown as for each species one transition is very low in intensity due to a dominant ligand-orbital contribution and correspondingly very low cross section (the plots of all HOMO, HOMO-1... orbitals, including the ligand-dominated ones, can be found in Figures S13 and S14).

Table S5: RIXS transitions for Cp*Rh$(CO)_2$.

Cp*Rh$(CO)_2$				
Energy / eV	Initial orbital	Final orbital	Contribution / %	Transition
3.0	73	74	98.3	HOMO $\rightarrow$ LUMO
4.0	72	74	93.8	HOMO-1 $\rightarrow$ LUMO
4.9	70	74	77.9	HOMO-3 $\rightarrow$ LUMO
5.4	69	74	63.8	HOMO-4 $\rightarrow$ LUMO
5.6	68	74	60.0	HOMO-5 $\rightarrow$ LUMO

Table S6: RIXS transitions for Cp*Rh(CO)-oct.

Cp*Rh(CO)-oct.				
Energy / eV	Initial orbital	Final orbital	Contribution / %	Transition
2.1	99	100	97.3	HOMO $\rightarrow$ LUMO
3.1	98	100	94.2	HOMO-1 $\rightarrow$ LUMO
3.6	97	100	64.4	HOMO-2 $\rightarrow$ LUMO
4.0	95	100	56.9	HOMO-4 $\rightarrow$ LUMO
4.8	94	100	73.1	HOMO-5 $\rightarrow$ LUMO

Table S7: RIXS transitions for CpRh$(CO)_2$.

CpRh$(CO)_2$				
Energy / eV	Initial orbital	Final orbital	Contribution	Transition
3.2	53	54	97.9	HOMO $\rightarrow$ LUMO
4.0	52	54	97.1	HOMO-1 $\rightarrow$ LUMO
4.9	51	54	52.6	HOMO-2 $\rightarrow$ LUMO
5.4	49	54	42.6	HOMO-4 $\rightarrow$ LUMO
6.0	48	54	54.8	HOMO-5 $\rightarrow$ LUMO

Table S8: RIXS transitions for CpRh(CO)-oct.

CpRh(CO)-oct.				
Energy / eV	Initial orbital	Final orbital	Contribution	Transition
2.2	79	80	97.5	HOMO → LUMO
3.1	78	80	96.6	HOMO-1 → LUMO
3.5	77	80	93.6	HOMO-2 → LUMO
4.0	76	80	89.6	HOMO-3 → LUMO
5.1	74	80	58.9	HOMO-5 → LUMO

S2.3.3 Calculated MOs

The LUMOs of $Cp^*Rh(CO)_2$, $CpRh(CO)_2$, $Rh(acac)(CO)_2$, as well as their respective σ-complexes are shown in Figure S11.

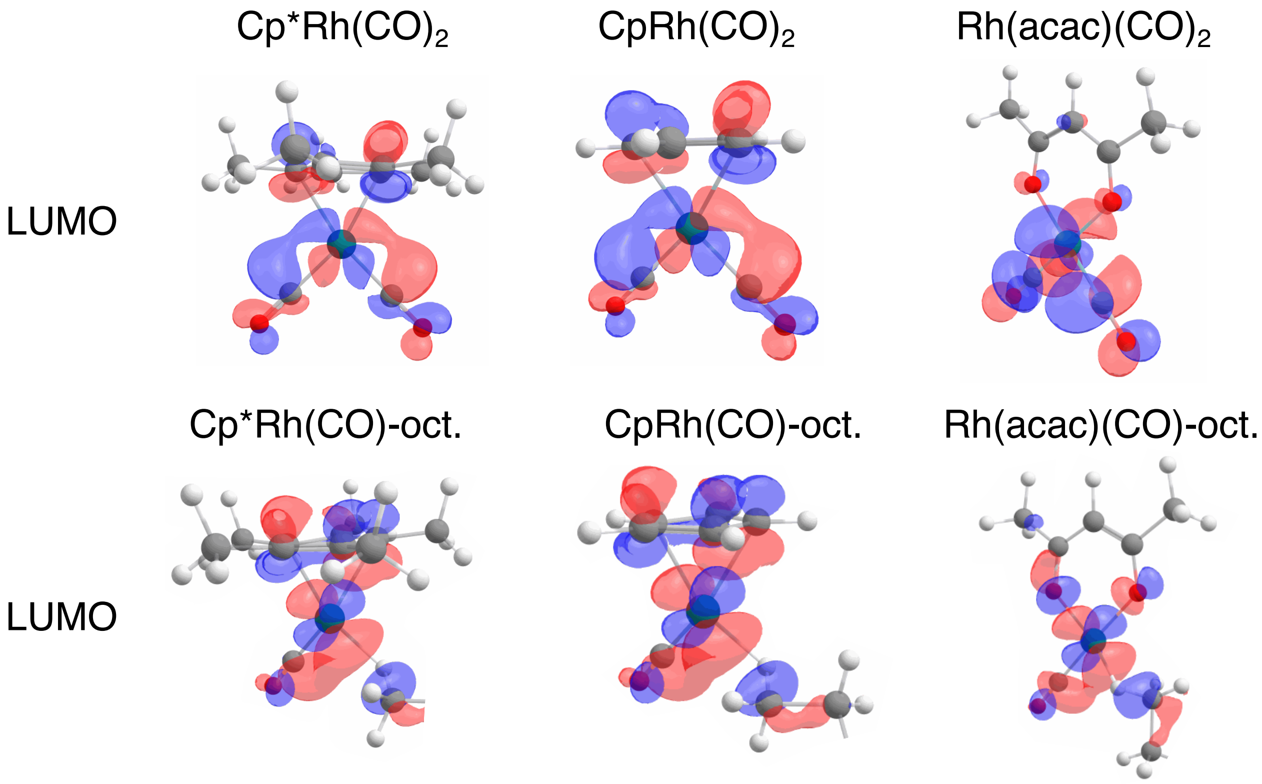

Figure S11: Calculated LUMOs of $Cp^*Rh(CO)_2$, Cp*Rh(CO)-octane, $CpRh(CO)_2$, CpRh(CO)-octane, $Rh(acac)(CO)_2$ and Rh(acac)(CO)-octane. All MOs are shown with an isovalue of 0.043.

The occupied MOs of the two dicarbonyl complexes $Cp^*Rh(CO)_2$ and $CpRh(CO)_2$, as well as for the corresponding σ-complexes are shown in Figure S12 and S13, respectively.

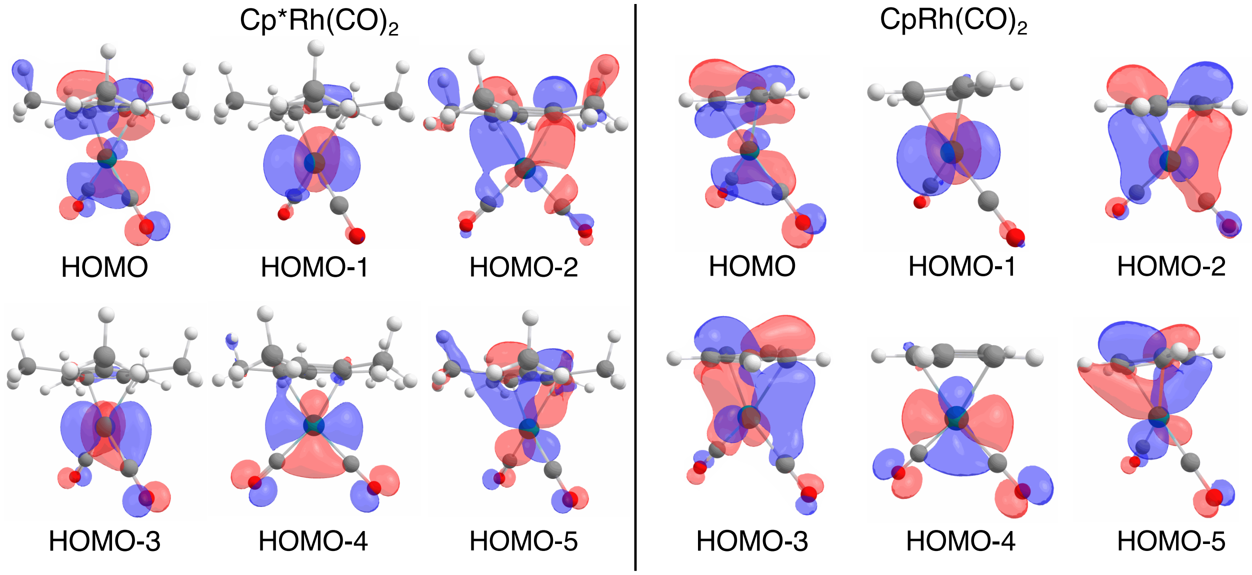

Figure S12: Calculated occupied molecular orbitals of $Cp^*Rh(CO)_2$ and $CpRh(CO)_2$. All MOs are shown with an isovalue of 0.043.

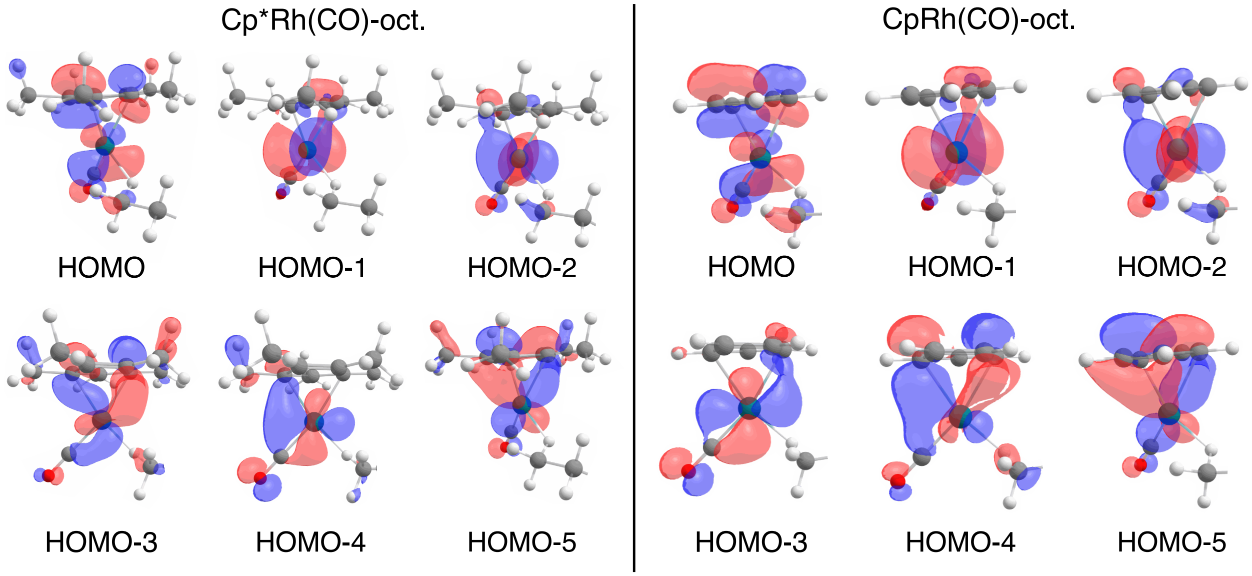

Figure S13: Calculated occupied molecular orbitals of Cp*Rh(CO)-octane and CpRh(CO)-octane. All MOs are shown with an isovalue of 0.043.

Figure S14 shows the HOMO orbital of Cp*Rh(CO)-octane and CpRh(CO)-octane with a lower isovalue to improve the visibility of the phase at the C-H bond coordinating to the Rh atom.

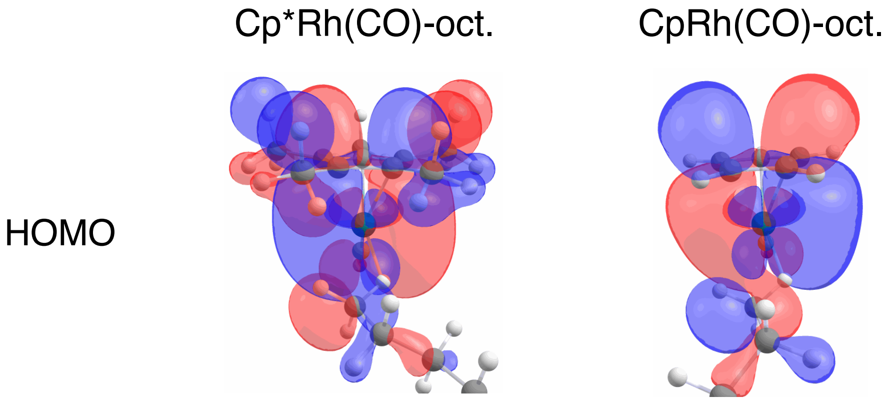

Figure S14: Calculated HOMOs of Cp*Rh(CO)-octane and CpRh(CO)-octane. Both MOs are shown with an isovalue of 0.01.

S2.3.4 Rh 4d character of occupied MOs

Table S9 and S10 show the Rh 4d character for the MOs presented in Figure S12 and S13.

Table S9: Calculated molecular orbital nature and Rh 4d character of the dominating occupied molecular orbitals determining the valence electronic structures and the calculated RIXS spectra of the $CpRh(CO)_2$ and $Cp^*Rh(CO)_2$ (HOMO-2 and HOMO-3 energies and names are flipped in the two dicarbonyl-complexes).

	$Cp^*Rh(CO)_2$		$CpRh(CO)_2$	
MO nature	Name	Rh 4d char. / %	Name	Rh 4d char. / %
$4d_{yz} + \pi^*(CO)$	HOMO	15.5	HOMO	22.5
$4d_{z^2}$	HOMO-1	75.9	HOMO-1	78.0
$4d_{xz}$	HOMO-3	81.4	HOMO-2	64.1
$\pi(Cp^*/Cp)$	HOMO-2	12.5	HOMO-3	29.3
$4d_{x^2-y^2}$	HOMO-4	61.2	HOMO-4	67.7
$4d_{yz} + \pi^*(Cp^*/Cp)$	HOMO-5	51.6	HOMO-5	47.9

Table S10: Calculated molecular orbital nature and Rh 4d character of the dominating occupied molecular orbitals determining the valence electronic structures and the calculated RIXS spectra of the CpRh(CO)-octane and Cp*Rh(CO)-octane σ-complexes (HOMO-3 and HOMO-4 energies and names are flipped in the two σ-complexes).

	Cp*Rh(CO)–octane		CpRh(CO)–octane	
MO nature	Name	Rh 4d char. / %	Name	Rh 4d char. / %
$4d_{yz} + \pi^*(CO)/\sigma^*(C-H)$	HOMO	29.3	HOMO	38.1
$4d_{z^2}$	HOMO-1	71.9	HOMO-1	77.0
$4d_{xz}$	HOMO-2	80.0	HOMO-2	80.8
$\pi(Cp^*/Cp)$	HOMO-3	34.2	HOMO-4	21.5
$4d_{x^2-y^2}$	HOMO-4	65.5	HOMO-3	72.9
$4d_{yz} + \pi^*(Cp^*/Cp)$	HOMO-5	47.1	HOMO-5	41.6

Additionally the contribution of the coordinating C-H bond to the HOMO was calculated to be 2.7% for Cp*Rh(CO)-octane and 3.2% for CpRh(CO)-octane, matching the notion that in the case of theCp* Rh $4d_{yz}$ orbital mixes to a larger extent with the coordinating C-H bond.

S2.3.5 Discussion of RIXS transitions in Cp*Rh(CO)$_2$ and CpRh(CO)$_2$

In this section, the underlying RIXS peaks in the calculated spectra of Cp*Rh(CO)$_2$ and CpRh(CO)$_2$ (see Figure S10 top), are discussed in greater detail. We start the analysis with the spectrum of Cp*Rh(CO)$_2$ (see Figure 4a, main text). The first peak in the Cp*Rh(CO)$_2$ RIXS spectrum at an energy transfer of 3.0 eV corresponds to the transition of electrons from the highest occupied molecular orbital (HOMO), which is derived from the 4d$_{yz}$ orbital, to the LUMO (see Table S5). The intensity of this transition is lower than that of the other main transitions, which can be attributed to the lower Rh 4d character in the HOMO (15.5% according to our calculations, see Table S9). The small Rh 4d character is due to strong orbital mixing with ligand-based orbitals, including a π-orbital of the Cp*-ring and the π^*-orbitals of the CO ligands (see a plot of all MOs in Figure S12) which results in a low oscillator strength in the HOMO $\rightarrow$ Rh 2p step of the RIXS process. The main interaction in the HOMO can be classified as the back-donation of the Rh 4d$_{yz}$ orbital to the CO π^* orbitals.

The next higher transition occurs at an energy transfer of 4.0 eV and can be assigned to the HOMO-1 $\rightarrow$ LUMO transition (see Table S5). HOMO-1 is derived from the 4d$_{z^2}$ orbital with pronounced atomic character (Rh 4d character of 75.1%, see Table S9) and, accordingly, a stronger RIXS transition compared to HOMO. In line with this trend, at 4.9 eV and 5.4 eV we find HOMO-3 and HOMO-4 $\rightarrow$ LUMO transitions, respectively. HOMO-3 and HOMO-4 are derived from the 4d$_{xz}$ and 4d$_{x^2-y^2}$ orbitals with 81.4% and 61.2% Rh 4d character, respectively. The last dominant RIXS transition appears at 5.6 eV and relates to HOMO-5 $\rightarrow$ LUMO transitions, where the HOMO-5 can be characterized as π-bonding interaction between an occupied π-orbital of the Cp*-ring and the Rh 4d$_{yz}$ orbital (as discussed in detail in main manuscript for the HOMO-5 in the corresponding σ-complex).

The features and trends observed in CpRh(CO)$_2$ closely resemble those in its methylated analogue (see Figure 4b, main text). The occupied MOs in CpRh(CO)$_2$ have the same nature as in Cp*Rh(CO)$_2$, with small energy changes (Table S7) and Rh 4d character (Table

S9). Accordingly, the RIXS transitions from these mostly atomic-like MOs ($4d_{z^2}$-, $4d_{xz}$-, and $4d_{x^2-y^2}$-derived orbitals) to the LUMO appear at the same energy transfer as in their methylated counterpart. The most significant effect of methylating the cyclopentadienyl ring is a shift in the energetic position of the HOMO → LUMO and HOMO-5 → LUMO transitions, which correspond to the RIXS peaks at lowest and highest energy transfer in the RIXS spectra (Figure S10, top), respectively. In $CpRh(CO)_2$ these transitions appear at energy transfers of 3.2 eV (HOMO-5 → LUMO) and 6.0 eV (HOMO-5 → LUMO) while in $Cp^*Rh(CO)_2$ the corresponding transition energies are 3.0 eV and 5.6 eV (compare Tables S7 and S5). This suggests a slight destabilization of both HOMO and HOMO-5 in $Cp^*Rh(CO)_2$ compared to $CpRh(CO)_2$. The Rh 4d character of the HOMO is slightly higher in $CpRh(CO)_2$ (22.5% compared to 15.5% in $Cp^*Rh(CO)_2$, see Table S9), indicating a weaker hybridization with the π-orbital of the Cp-ring and the π^*-orbitals of the CO ligands. The methylated, more electron-rich Cp* ring, allows for stronger orbital mixing with the $4d_{yz}$ orbital and the π^*-orbital of the CO ligand, thereby lowering the 4d character. The opposite trend is observed for the HOMO-5, which exhibits a lower 4d character of 47.9% in $CpRh(CO)_2$ (as opposed to 51.6% in $Cp^*Rh(CO)_2$, see Table S9). A higher electron density in the Cp* ring leads to a decreased delocalization of electron density of the Rh $4d_{yz}$-derived orbitals onto the Cp* ligand, which destabilizes the orbital in energy, weakens the degree of orbital mixing, and shifts the HOMO-5 → LUMO transition to lower energy transfers.

S2.3.6 Discussion of atomic like RIXS transitions in Cp*Rh(CO)-octane and CpRh(CO)-octane

As the most relevant RIXS transitions in Cp*Rh(CO)-octane and CpRh(CO)-octane have been discussed in great detail in the main manuscript, we focus here on the mostly atomic like MOs derived by Rh 4d$_{z^2}$, 4d$_{xz}$, 4d$_{x^2-y^2}$ and their RIXS transitions. All three MOs do not play a major role in determining bonding between the C-H bond and the Rh center, due to a symmetry mismatch with the alkane ligand. Their peaks appear at nearly the same energy transfers for both Cp*Rh(CO)-octane and CpRh(CO)-octane (see Table S6 and Table S8), which is a consequence of their mostly atomic-like character. Due to the symmetry mismatch with the C-H σ-orbital, these Rh-centered orbitals do not experience a stabilization by coordination of the alkane. This effect is particularly pronounced for MOs with significant contributions from the CO π*-orbital in the dicarbonyl complex, like the 4d$_{xz}$ and 4d$_{x^2-y^2}$ derived MOs. In the σ-complex, their reduced interaction with the CO π*-orbital decreases their stability, leads to a larger shift in the RIXS transition.

S2.3.7 Extraction of RIXS spectra and $CpRh(CO)_2$ vs $Rh(acac)(CO)_2$ comparison

For each species, a RIXS map was constructed based on the calculated RIXS transitions. The RIXS spectrum is then extracted by a cut at the desired incidence energy. The comparison of the measured time-resolved difference RIXS spectra of $CpRh(CO)_2$ and $Rh(acac)(CO)_2$ at a pump-probe delay of 25 ps and at an incidence energy of 3004.2 eV or 3004.4 eV, respectively, is shown in Figure S15.

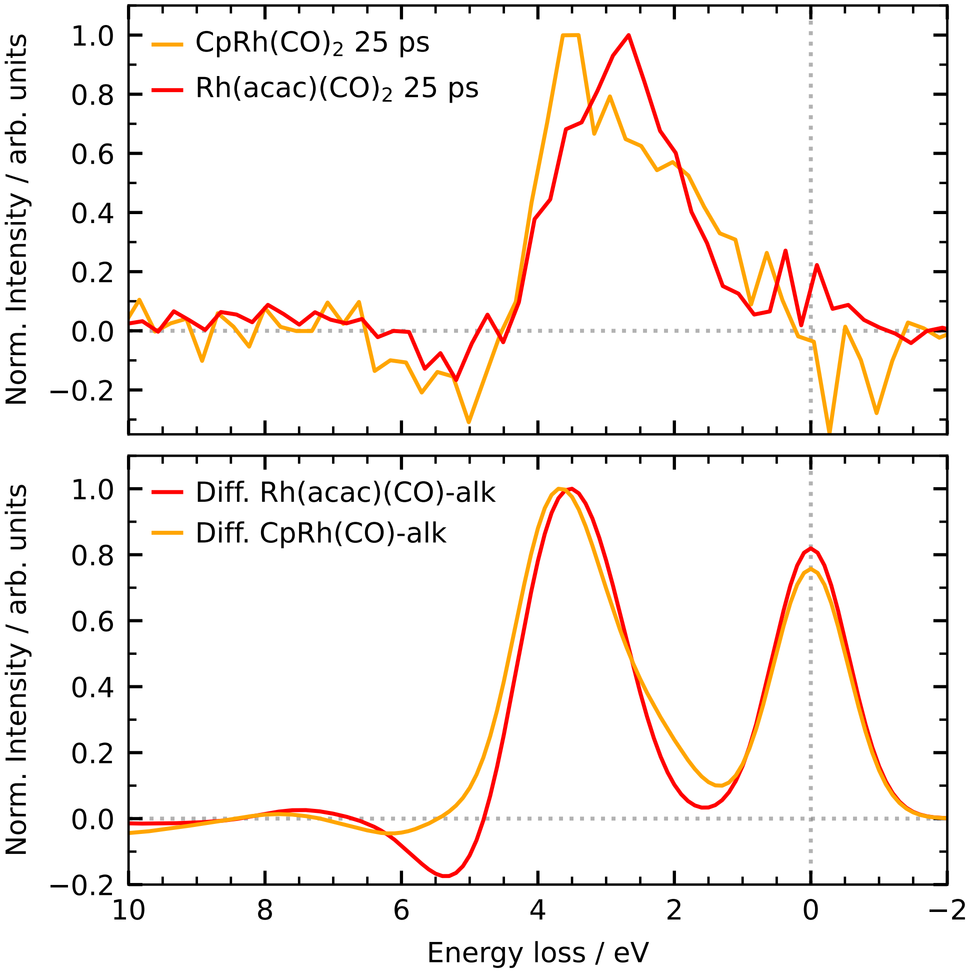

Figure S15: (Top) Experimental time-resolved difference Rh L_3-edge RIXS spectra of $CpRh(CO)_2$ and $Rh(acac)(CO)_2$ at a pump-probe delay of 25 ps and at an incidence energy of 3004.2 eV or 3004.4 eV, respectively. (Bottom) Calculated difference spectra of the respective σ-complex.

The general trends observed in the experimental spectra (discussion in the main manuscript) are also visible in the calculated spectra. However, the agreement of the theoretical RIXS spectra of $Rh(acac)(CO)_2$ with the experimental spectra is worse than the agreement of $Cp^{*}Rh(CO)_2$ and $CpRh(CO)_2$. We are currently investigating the reason for this including potential relations to the differences in the nature of the bonds in the respective systems. In the main text, the difference RIXS spectra of $Cp^{*}Rh(CO)_2$ and $CpRh(CO)_2$ are compared by interpolating the spectrum of $CpRh(CO)_2$ using the energy transfer axis of $Cp^{*}Rh(CO)_2$. To verify this approach, the opposite variant of this interpolation (interpolating the spectrum of $Cp^{*}Rh(CO)_2$ using the energy transfer axis of $CpRh(CO)_2$), is shown in direct comparison in Figure S16. No significant differences in the resulting spectra is observed.

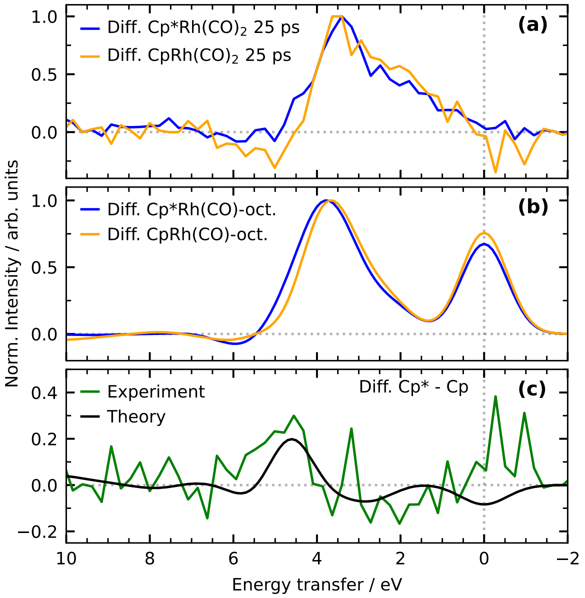

(a) The spectrum of $Cp^{*}Rh(CO)_2$ was interpolated using the energy transfer axis of $CpRh(CO)_2$ to get a common x-axis.

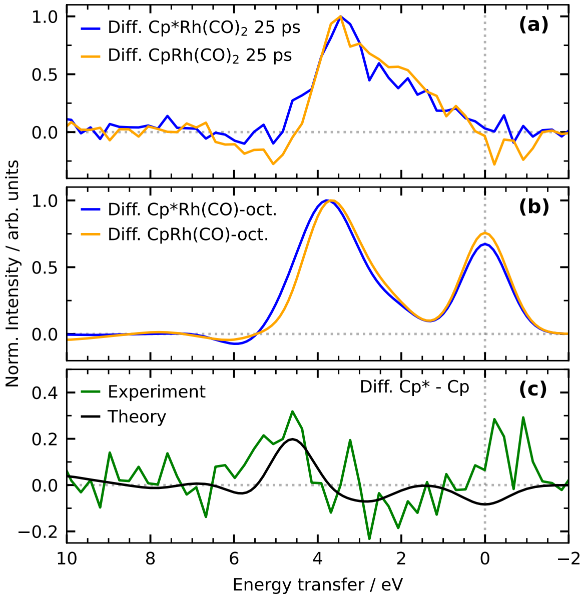

(b) The spectrum of $CpRh(CO)_2$ was interpolated using the energy transfer axis of $Cp^{*}Rh(CO)_2$ to get a common x-axis.

Figure S16: In both subplots: (a) Time-resolved difference Rh L_3-edge RIXS spectra of $Cp^{*}Rh(CO)_2$ and $CpRh(CO)_2$ at a pump-probe delay of 25 ps at an incidence energy of 3004.2 eV. (b) Calculated difference RIXS spectra of CpRh(CO)-octane and Cp*Rh(CO)-octane at the same incidence energy as the experimental spectra. All difference spectra are normalized to the maximum of their inelastic feature. (c) Difference of experimental and calculated difference spectra.

S2.3.8 Energy landscape and activation barrier

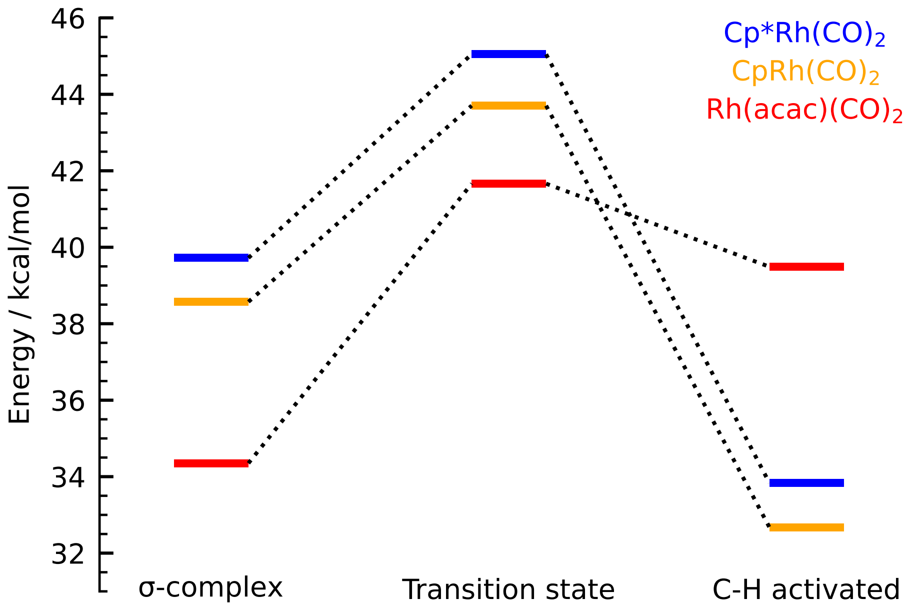

Figure S17: Calculated free energy landscapes of the octane σ-complex, transition state and C-H activated species for $Cp^*Rh(CO)_2$, $CpRh(CO)_2$, and $Rh(acac)(CO)_2$. The free energies are calculated with respect to the stable reactants (i.e., $Cp^*Rh(CO)_2/CpRh(CO)_2/Rh(acac)(CO)_2$ + octane).

S2.3.9 Advanced Fragment Decomposition analysis

As mentioned in the main text the sigma complexes Cp*Rh(CO)-octane and CpRh(CO)-octane can be schematically constructed from the Cp*/Cp and Rh^+(CO)-octane fragments. Tables S11 and S12 show a detailed decomposition of the HOMO and HOMO-5 of Cp*Rh(CO)-octane and CpRh(CO)-octane based on the above-mentioned fragments.

Table S11: Advanced fragment decomposition analysis of the HOMO and HOMO-5 of Cp*Rh(CO)-octane.

Cp*Rh(CO)-octane		
HOMO		
Contribution / %	Character Cp*	Character Rh^+(CO)-oct.
47.3	π^*-orbital	-
16.7	-	Rh 4d_{yz} + π^*(CO)
15.7	-	Rh 4d_{yz} + σ^*(C-H)
HOMO-5		
Contribution / %	Character Cp*	Character Rh^+(CO)-oct.
37.1	π^*-orbital	-
42.0	-	Rh 4d_{yz} + π^*(CO)
7.2	-	Rh 4d_{yz} + σ^*(C-H)

Table S12: Advanced fragment decomposition analysis of the HOMO and HOMO-5 of CpRh(CO)-octane.

CpRh(CO)-octane		
HOMO		
Contribution / %	Character Cp	Character Rh^+(CO)-oct.
40.1	π^*-orbital	-
16.9	-	Rh 4d_{yz} + π^*(CO)
24.7	-	Rh 4d_{yz} + σ^*(C-H)
HOMO-5		
Contribution / %	Character Cp	Character Rh^+(CO)-oct.
46.5	π^*-orbital	-
32.0	-	Rh 4d_{yz} + π^*(CO)
11.4	-	Rh 4d_{yz} + σ^*(C-H)

Analogous to the fragmentation charge decomposition analysis in the main manuscript, the same analysis can be repeated in the dicarbonyl complexes. Here, the fragments Cp*/Cp and $Rh^+(CO)_2$ are considered. The results are shown in Table S13. The Cp* ligand donates 0.09 electrons more to $Rh^+(CO)_2$ than the Cp ligand. This trend is consistent with the one observed in the σ-complexes.

Table S13: Fragment decomposition analysis of $Cp^*/CpRh(CO)_2$.

Complex	Electronic charge transfer (in e^-) $Cp^*/Cp \rightarrow Rh^+(CO)_2$
$Cp^*Rh(CO)_2$	0.89
$CpRh(CO)_2$	0.80

References

(1) Jay, R. M.; Banerjee, A.; Leitner, T.; Wang, R.-P.; Harich, J.; Stefanuik, R.; Wikmark, H.; Coates, M. R.; Beale, E. V.; Kabanova, V.; others Tracking C–H activation with orbital resolution. *Science* **2023**, *380*, 955–960.

(2) Trejo, L.; Trejo, L. Vand 2 were calcd. from the. *Selected Data on Mixtures: Thermodynamic properties of non-reacting binary systems of organic substances* **1991**, 30.

(3) National Center for Biotechnology Information PubChem Compound Summary for CID 356, n-Octane. 2026; https://pubchem.ncbi.nlm.nih.gov/compound/356, Accessed: 2026-02-26.

(4) Cannelli, O.; Bacellar, C.; Ingle, R.; Bohinc, R.; Kinschel, D.; Bauer, B.; Ferreira, D.; Grolimund, D.; Mancini, G.; Chergui, M. Toward time-resolved laser T-jump/X-ray probe spectroscopy in aqueous solutions. *Structural Dynamics* **2019**, *6*.

(5) Jay, R. M.; Coates, M. R.; Zhao, H.; Winghart, M.-O.; Han, P.; Wang, R.-P.; Harich, J.; Banerjee, A.; Wikmark, H.; Fondell, M.; others Photochemical Formation and Electronic Structure of an Alkane σ-Complex from Time-Resolved Optical and X-ray Absorption Spectroscopy. *Journal of the American Chemical Society* **2024**, *146*, 14000–14011.
